# Seasonal thawing of high Arctic soils triggers selective microbial growth and predation

**DOI:** 10.1128/msystems.00738-25

**Published:** 2026-05-07

**Authors:** Margaret A. Cramm, Ömer K. Coskun, Francesco Montemagno, Matteo Selci, Daniel S. Read, Tim Goodall, Brianna Green, Sayali A. Mulay, Katie Sipes, Andrey A. Abramov, Catherine M. Heppell, Julia Boike, Donato Giovannelli, Tatiana A. Vishnivetskaya, Robert L. Hettich, Andrew D. Steen, Karen G. Lloyd, William D. Orsi, Anne D. Jungblut, James A. Bradley

**Affiliations:** 1 School of Geography, Queen Mary University of London, London, United Kingdom; 2 School of Biological and Behavioural Sciences, Queen Mary University of London, London, United Kingdom; 3 Department of Science, Natural History Museum, London, United Kingdom; 4 Department of Earth and Environmental Sciences, Paleontology & Geobiology, Ludwig-Maximilians-Universität München, Munich, Germany; 5 Department of Biology, University of Naples Federico II 9307, Naples, Italy; 6 Department of Marine and Coastal Sciences, Rutgers University, New Brunswick, New Jersey, USA; 7 UK Centre for Ecology & Hydrology 41865, Wallingford, United Kingdom; 8 Department of Earth and Planetary Sciences, University of Tennessee 4292, Knoxville, Tennessee, USA; 9 Department of Microbiology, University of Tennessee 4292, Knoxville, Tennessee, USA; 10 Biosciences Division, Oak Ridge National Laboratory 6146, Oak Ridge, Tennessee, USA; 11 Kovda Institute of Physicochemical and Biological Problems in Soil Science, Russian Academy of Sciences, Pushchino, Russia; 12 Chilterns National Landscape, Chinnor, United Kingdom; 13 Permafrost Research, Alfred Wegener Institute Helmholtz Centre for Polar and Marine Research, Potsdam, Germany; 14 Geography Department, Humboldt-Universität of Berlin 9373 , Berlin, Germany; 15 Institute for Marine Biological Resources and Biotechnologies, Italian National Research Council, CNR-IRBIM, Ancona, Italy; 16 Earth-Life Science Institute, ELSI, Tokyo Institute of Technology, Tokyo, Japan; 17 Marine Chemistry and Geochemistry Department, Woods Hole Oceanographic Institution, Woods Hole, Massachusetts, USA; 18 Department of Biological Sciences, University of Southern California, Dornsife College of Letters, Arts and Sciences 5116, Los Angeles, California, USA; 19 Department of Earth Sciences, University of Southern California, Dornsife College of Letters, Arts and Sciences 5116, Los Angeles, California, USA; 20 GeoBio-Center LMU, Ludwig-Maximilians-Universität München, Munich, Germany; 21 Aix Marseille Univ, Université de Toulon, CNRS, IRD, MIO, Marseille, France; Pacific Northwest National Laboratory, Richland, Washington, USA; Pacific Northwest National Laboratory, Richland, Washington, USA; The University of Melbourne, Melbourne, Victoria, Australia

**Keywords:** Arctic soil, microbial communities, carbon cycle, qSIP, microbial dormancy

## Abstract

Climate warming threatens Arctic permafrost with seasonal cycles of freezing and thawing. Arctic soil microorganisms regulate carbon stocks and greenhouse gas exchanges with the atmosphere, yet their precise seasonal growth and dormancy dynamics, and their responses to permafrost thaw, are not well understood. We thawed frozen Svalbard active layer soil and traced microbial growth using DNA quantitative stable isotope probing with H_2_^18^O. We observed temporal growth patterns resulting in distinct early (21-day) and late-stage (98-day) growing microbial populations. In particular, Acidobacteriota, Actinobacteriota, Bacteroidota, Proteobacteria, and predatory and epibiont bacterial taxa (such as those affiliated to Bdellovibrionota and Patescibacteria) were identified in the soil active layer as clades that were growing following thawing. Methane concentrations in our microcosms remained low, yet *pmoA* genes were ^18^O-labeled, indicating growth of aerobic methane-oxidizing bacteria. Approximately half of the microbial taxa detected did not grow, suggesting that Arctic soils constitute sizeable reservoirs of dormant microorganisms. Our results reveal complex and temporal microbial dormancy, growth, death, predation, and parasitism dynamics in seasonally changing Arctic soils. These processes likely regulate the exchange and storage of soil carbon across the increasingly vulnerable Arctic region.

**IMPORTANCE:**

Microorganisms play key roles in transforming soil carbon into greenhouse gases. As Arctic soils warm as a result of climate change, greater depths and expanses of permanently frozen soil are experiencing seasonal thaw. Despite the importance of active soil microorganisms in transforming soil carbon, the seasonal freezing and thawing of Arctic soils and associated dormancy and re-activation of microbial populations are not well constrained. Here, we thawed and incubated active layer (i.e., seasonally thawing) Arctic soil with a stable isotope to directly label the DNA of growing soil microorganisms. We found that half of the microbial diversity did not grow after thaw and that some groups, including the Bacteroidota and predatory bacteria, grew disproportionately. The growing microbial community shifted over time, and bacteria capable of oxidizing methane grew more after prolonged thaw. These findings highlight that dormancy, predation, and variable growth dynamics are important factors determining ecological and biogeochemical processes in thawing Arctic soil.

## INTRODUCTION

Arctic soils contain approximately a third of the global soil carbon (C) pool (1, 2) and are at the forefront of climate change, since air temperatures in the Arctic are warming four times faster than the global average (3). As a consequence, Arctic permafrost is thawing, and the permafrost active layer (which experiences seasonal freezing and thawing) is deepening and exposing vast carbon stocks to microbial degradation (4–6). Permafrost thaw is predicted to release 5%–15% of Arctic soil carbon as carbon dioxide (CO_2_) and methane (CH_4_) by 2100 under the current global trajectory (RCP8.5) (2, 7), causing a 0.13°C–0.27°C increase in global average surface temperature (7).

The production of greenhouse gases from active layer soils and permafrost is a microbially driven process (8, 9). With increasing permafrost thawing depth and duration, soil microorganisms in newly formed active layer soils will be able to degrade a larger source of carbon substrates for longer and potentially at faster rates, thus increasing soil respiration and greenhouse gas emissions to the atmosphere (10).

Microbially mediated transformation of carbon from soils to the atmosphere is dependent on the composition and metabolic capabilities of the organisms inhabiting the soil environment (11, 12), as well as the physico-chemical characteristics of the soil, and the physical and biochemical actions of plants and animals in the soil and on its surface (8, 13–20). Active layer microbial communities exhibit seasonal variations in the relative abundances of certain bacterial phyla (21, 22), with Proteobacteria, Acidobacteriota, Actinobacteriota, and Verrucomicrobiota often found representing dominant portions of the community (22–25). Schostag et al. (26) effectively showed changes in bacterial, fungal, and protozoan responses to thaw in Svalbard active layer soil using RNA sequencing, showing dominance of copiotrophic bacteria in the first 16 days following thaw. This study demonstrated the utility of physiology-based genomic investigations for understanding microbial carbon degradation in Arctic soil. Nevertheless, the seasonal changes in microbial dynamics in Arctic soils, including growth, death, and dormancy, are not well understood, despite the important role that microbial physiology plays in soil carbon turnover and the need to capture and constrain these processes in soil microbial and biogeochemical models (27–31). Pulses of greenhouse gases measured following Arctic soil active layer thawing highlight that the microbial community is quick to respond to thaw, with important consequences on greenhouse gas fluxes from Arctic terrestrial environments (32). However, despite the critical connection between active layer soil microorganisms and the global carbon budget, we lack data on the precise population dynamics and microbe-associated carbon transformations in Arctic active layer soils following thawing due to seasonal changes.

Quantitative DNA stable isotope probing (qSIP) with H_2_^18^O is an effective physiology-based genomic method for measuring taxon-specific growth, death, and dormancy (33). qSIP has clarified microbial growth dynamics in experimentally warmed tundra soils, showing that the length of warming (months versus decades) affects clade-specific microbial growth and bacterial growth rates (34). qSIP was further used to reveal taxon-specific growth rates of experimentally warmed Antarctic glacial forefield soils that were sensitive to successional stage and local vegetation (35).

To understand the changes in microbial community dynamics, including the growth and activity of microorganisms in active layer soil following thaw, we conducted *ex situ* incubations of subsurface active layer soils from Svalbard, in the high Arctic, one of the regions presently facing the fastest rates of warming on Earth (36, 37). Our aim was to identify growing and non-growing microorganisms in active layer soil at two time points following thaw (21 and 98 days) and to identify associated taxon-specific changes in microbial community growth.

## MATERIALS AND METHODS

### Study site and sample collection

**Fig 1:**
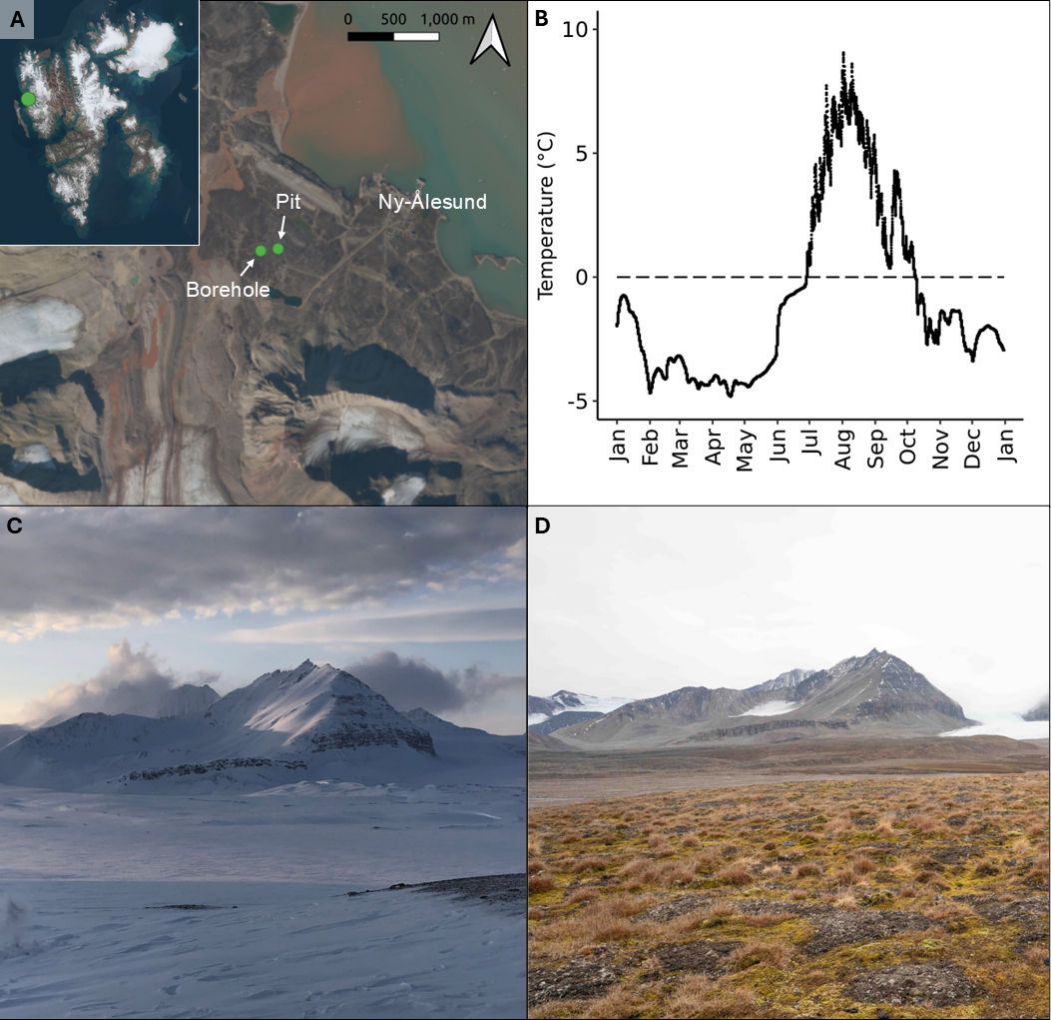
Location, annual soil temperature, and photographs of the Bayelva Permafrost Monitoring Observatory near Ny-Ålesund, Svalbard. (**A**) Map showing the location of the core borehole (78.921078° N, 11.857037° E) and the soil pit (78.921367° N, 11.865867° E). Map data were obtained from the Norwegian Polar Institute. (**B**) Soil temperature at the Bayelva Permafrost Monitoring Observatory near Ny-Ålesund, Svalbard, at 21 cm depth from 1 January 2021 to 2 January 2022. Soil temperature data are from Boike et al. (39) and correspond to soil temperature at 21 cm beneath the soil surface. The dashed horizontal line indicates 0°C. The seasonal characteristics of the ground near the observatory can be seen in panels C and D. Pictures were taken in (**C**) March 2022 and (**D**) September 2022 by Margaret Cramm.

Our study site, the Bayelva Permafrost Monitoring Observatory near Ny-Ålesund (78.921078° N, 11.857037° E), is situated on the Brøggerhalvøya peninsula in northwest Svalbard. The area is warmed by the North Atlantic’s West Spitsbergen Current and has an average air temperature range of −17.0°C to −3.8°C in January and 4.6°C to 6.9°C in July (between 1993 and 2011) (38). The Bayelva area is underlain by continuous permafrost that is ~100 m thick, and the depth of the active layer is 1 to 2 m (38). The site is covered in bare non-sorted circles of 1 m diameter, surrounded by rims hosting grasses, sedges, lichens, and moss (38), and the soil is of mineral type (13). In 2021, the year of sampling, the range of temperature of the soil at a depth of 21 cm was −4.8°C to 9.1°C, with 104 continuous days of temperatures above 0°C in 2021 and 59 continuous days above 4°C (Fig. 1B).

On 25 March 2021, we extracted a single frozen core from the soil surface to 2.4 m depth from Bayelva Permafrost Monitoring Observatory near Ny-Ålesund (78.921078° N, 11.857037° E) (Fig. 1) using a dry borehole method as described in Shi et al. (40). We subsampled subsurface active layer soil from this core, extracting soil from a depth corresponding to 18–27 cm below the soil surface. We transferred the loose, mixed material into sterile Whirl-Pak bags and stored them at −20°C for 3 months before incubation at 4°C to simulate seasonal thawing. Methods for obtaining soil moisture, organic matter content, and cell abundance from the soil core are described in the Supplemental Methods. On 18 June 2022, we collected soil from a nearby 35 cm excavated rectangular soil pit (78.921367° N, 11.865867° E) (41) that maintained an intact wall. We stored this soil in a Whirl-Pak bag at −20°C until analyses. This soil was analyzed for trace element concentrations to serve as a general reference for the geochemical characteristics of soils at the Bayelva site. Methods for obtaining trace element data from the soil pit are described in the Supplemental Methods.

### Microcosm set-up

To create microcosms of thawing active layer soil, we thawed soil collected from 18 to 27 cm depth of the frozen core at room temperature and immediately placed 2 g of soil into autoclaved 20 mL glass vials on ice. We added 1 mL of either heavy water containing the 97% ^18^O isotope of oxygen (H_2_^18^O), or water of natural isotopic composition (H_2_^16^O, ultra-pure Milli-Q water, autoclaved and filter-sterilized through a 0.2 µm pore-size filter). Microorganisms growing in the presence of H_2_^18^O incorporate ^18^O into newly synthesized DNA molecules at a rate equivalent to DNA incorporation of ^16^O from H_2_^16^O (42). The headspace in each individual vial after the addition of soil and water was approximately 18 mL. We created 10 replicate microcosms: 5 intended for a 21 day incubation and 5 for a 98 day incubation. In 2021, soil from this site and depth was above 0°C for 104 days; thus, the longer 98 day incubations correspond roughly to the duration of seasonal thawing. Eight replicate microcosms (four for the 21 day incubation and four for the 98 day incubation groups) received water of standard (untreated) isotopic composition (>99% H_2_^16^O). Two replicate microcosms (one for each of the 21 and 98 day incubation groups) received H_2_^18^O. The microcosm replication for DNA SIP methodology was aligned with established methods (43). The water content of the 2:1 wt/vol ratio slurries contained 81% ^18^O isotope due to the dilution of the H_2_^18^O with natural soil moisture. This led to a five times increase in soil moisture in microcosms compared to the soil moisture of the collected soil, which is slightly higher than the typical three times soil moisture increase that occurs seasonally *in situ* as soils thaw during late spring/early summer (44). We sealed vials with butyl stoppers and exchanged the headspace with standardized zero-grade air (21% O_2_ with N_2_ balance, 0% CH_4_, 0% CO_2_). We created parallel anoxic microcosms exactly as above; the headspaces of these were flushed with zero-grade N_2_ gas. Microcosms were incubated at 4°C in the dark for 21 or 98 days. Following incubation, vials were frozen at −20°C.

### Headspace gas measurement

CO_2_, CH_4_, and nitrous oxide (N_2_O) were measured using gas chromatography with electron capture and flame ionization detection (Agilent Technologies 7890B GC system). For each measurement, 250 µL of air was removed (without replacement) from the headspace of incubation vials using a flame-sterilized gas-tight syringe and manually injected. Headspace gas was sampled on 0, 7, 14, 21, 28, 49, 77, and 98 days of incubation. A six-point calibration curve was created each day of gas measurement from a dilution series of calibration gas consisting of 100 ppm of CH_4_, 100 ppm of N_2_O, and 3,700 ppm of CO_2_. The headspace gas was not replaced during the incubation.

O_2_ was optically measured using a Fibox 4 Stand-alone Fiber Optic Oxygen Meter (PreSens Precision Sensing). Ethanol-sterilized 5 mm Planar Oxygen-Sensitive sensor spots (PreSens Precision Sensing GmbH) were mounted on the inside of three of five replicate vials before the incubation and prior to adding soil. O_2_ was measured (non-destructively) at least five times per week during the incubation.

### DNA extraction

DNA was extracted from vials as described in Coskun et al. (45) and further described in the Supplementary Methods. We purified the concentrated lysate using the Qiagen DNeasy PowerClean Pro Cleanup kit according to the manufacturer’s instructions and eluted the DNA in 100 µL. DNA was quantified using the fluorometric Qubit dsDNA HS Assay (Thermo Scientific) following the manufacturer’s instructions. DNA for unfractionated 16S rRNA gene amplicon sequencing libraries was diluted 1:1,000 with PCR-grade water to dilute PCR inhibitors that prevented PCR amplification of DNA in the eluted extraction, and in 1:10 and 1:100 dilutions. DNA for fractionation was not diluted prior to fractionation because this protocol diluted the DNA ~1:1,000. Two negative-control DNA extractions were done without soil or slurry added to the extraction mixture to assess potential levels of contamination.

### DNA fractionation

We fractionated DNA from the microcosm that received H_2_^18^O and a corresponding microcosm that received H_2_^16^O using density gradient centrifugation according to described DNA stable isotope probing methods (33, 45, 46). For three technical triplicates, 25 µL of DNA template was added to ~3.3 mL of cesium chloride (CsCl)-gradient buffer solution to a final density of 1.7 g mL^−1^ in 3.3 mL OptiSeal polyallomer tubes (Beckman Coulter). These tubes were centrifuged at 65,000 rpm for 72 h at 18°C in a TLN-100 Optima MAX-TL ultracentrifuge (Beckman Coulter) to create a density gradient. The density gradient was separated into 20 fractions (165 µL) within 3 h of centrifugation using a syringe pump and fraction recovery system as described in Coskun et al. (45) and in the video description in Cramm (47). The density of each fraction was inferred according to a linear density model based on the measured density of seven or eight fractions using an AR200 digital refractometer (Reichert Analytical Instruments). Fraction densities are shown in Fig. S1. We precipitated DNA from the density gradient using 2 µL glycogen (10 mg mL^−1^) and two volumes of filter- and UV-sterilized 30% polyethylene glycol 6000 solution (45, 46) and resuspended it in 30 µL PCR-grade water.

### Quantitative PCR (qPCR)

We quantified 16S ribosomal RNA (rRNA) genes from fractionated and unfractionated DNA with qPCR using the 515F-Y (5′-GTGYCAGCMGCCGCGGTAA-3′) and 806R (5′-GGACTACNVGGGTWTCTAAT-3′) primers (48, 49). The qPCR reaction mixture (20 µL) was dispensed using an epMotion 5070 pipetting robot (Eppendorf) and was composed of 4 µL template DNA, 0.4 µL of each primer (10 µM), 5.2 µL PCR-grade water, and 10 µL SsoAdvanced Universal SYBR Green Supermix (Bio-Rad). The PCR protocol was as follows: an initial denaturation at 95°C for 3 min, followed by 40 cycles of denaturation at 95°C for 10 s, followed by annealing and elongation at 55°C for 30 s, followed by fluorescence detection (CFX Connect real-time PCR system; Bio-Rad). qPCR reactions containing no template DNA were included in all reactions, and only samples for which the starting quantity (SQ) was more than the SQ for the “no-template” reaction were retained. Only qPCR runs with efficiency >90% and <110%, and *R*^2^ > 0.87, were retained.

We also quantified *pmoA* genes from fractionated and unfractionated DNA with qPCR using the A189F (5′-GGNGACTGGGACTTCTGG-3′) and Mb661R (5′-CCGGMGCAACGTCYTTACC-3′) primers (50), and *mcrA* genes from unfractionated DNA using the mlas (5′-GGTGGTGTMGGDTTCACMCARTA-3′) and mcrA-rev (5′-CGTTCATBGCGTAGTTVGGRTAGT-3′) primers (51). The qPCR reaction mix was the same as for qPCR of the 16S rRNA genes. The protocol for *pmoA* amplification was an initial denaturation at 94°C for 3 min, followed by 40 cycles of denaturation at 94°C for 30 s, annealing at 56°C for 40 s, and elongation at 72°C for 1 min, followed by fluorescence detection (StepOnePlus Real Time System, Applied Biosystems). The efficiency of the *pmoA* qPCR amplification was between 90 and 117% and *R*^2^ values were between 0.91 and 0.99. The protocol for *mcrA* amplification was an initial denaturation at 95°C for 3 min 30 s, followed by 40 cycles of denaturation at 95°C for 30 s, annealing at 55°C for 45 s, and elongation at 72°C for 30 s, followed by fluorescence detection (StepOnePlus Real Time System, Applied Biosystems). The efficiency and *R*^2^ for *mcrA* gene qPCR were 99% and 0.998, respectively.

### 16S rRNA gene sequencing and analysis

The V4 region of the 16S rRNA genes was amplified with polymerase chain reaction (PCR) using the 515F and 806R primers (48, 49). Triplicate PCR reactions were done using a touchdown protocol described in Cramm et al. (52) and further described in the Supplementary Methods. Negative PCR controls, in which no template DNA was added to the PCR reaction, were used to determine contamination during PCR. Negative DNA extraction controls and negative PCR controls were also prepared for sequencing to determine contamination. Triplicate PCR reactions were pooled and cleaned using AppMag PCR Clean Up Beads (Appleton) according to the manufacturer’s instructions. Equal moles of the pooled amplicons were sequenced on an Illumina MiSeq PE250 (2 × 250 bp) by the sequencing facility at the Natural History Museum (London, UK). 16S rRNA gene amplicon sequences can be found in the NCBI Sequence Read Archive under the BioProject accession PRJNA1118335.

Amplicon sequence variants (ASVs) were used for taxonomic community analysis. Adapters were removed from paired-end reads using Cutadapt version 4.0 (53), and reads shorter than 150 bp were discarded. We created ASVs using DADA2 version 1.16.0 (54, 55). We discarded reads with expected errors above 2. Forward and reverse reads were truncated to 250 bp and 200 bp, respectively, and reads shorter than these were discarded. The median library size was 51,343 reads (Fig. S2). Taxonomy was assigned using an RDP Naïve Bayesian Classifier algorithm and the SILVA v.138.1 sequence database (56, 57). Taxonomic names may differ from other sequence databases or published names (58). Diversity and statistical analysis were performed in R version 4.3.2 (59) using the packages vegan version 2.6-4 (60) and phyloseq version 1.46.0 (61). There were 2,666 ASVs in the data set with 40 singleton ASVs and 97 doubleton ASVs. We retained singletons and doubletons, and data were rarefied to 10,000 reads prior to alpha diversity calculations using Chao1 and Inverse Simpson indices. Indices were calculated based on the mean of 10 iterations. Diversity index data were evaluated for normality using the Shapiro-Wilk normality test (α = 0.05). Significance between the difference of means of normal diversity index data was determined using a Welch two-sample *t*-test (α = 0.05). Data were not rarefied, but they were transformed to relative sequence abundance for beta diversity calculations using Bray-Curtis distance matrix and visualization of 16S rRNA gene sequence community composition. The ANOSIM test (999 permutations, α = 0.05) was performed on the distance matrix to determine similarity of the different incubation groups.

### Estimating ^18^O enrichment of 16S rRNA genes

We calculated ^18^O-atom fraction excess (AFE) values according to methods described in Hungate et al. (33) and Coskun et al. (45, 62) using the HTSSIP package in R (63). For each technical replicate, AFE was calculated at the ASV level by the difference in density between the ASV in the H_2_^16^O control microcosm and its density in the corresponding H_2_^18^O microcosm. Only those ASVs that increased in density with 90% confidence after 1000 bootstraps and for which the lower confidence interval did not overlap zero were considered to have incorporated the ^18^O-label into their DNA. Negative labeling, in which the AFE is below 0 with a 90% confidence interval, occasionally arises due to density variability in rare ASVs (64) and can be disregarded. The technical replicates of the controls were analyzed separately to capture variability in the fractionating procedure. Due to a single technical replicate of the 98 day H_2_^16^O control group failing fractionation, only two technical H_2_^16^O control replicates were used to calculate AFE for the 98 day incubation. This may have caused wider 90% confidence intervals for the 98 day AFE values and therefore a possible increase in the number of ASVs showing a false negative result for ^18^O-labeling.

## RESULTS AND DISCUSSION

### Site and soil characteristics

We collected an active layer soil sample from near the Bayelva Permafrost Monitoring Observatory site (78.921078° N, 11.857037° E) near Ny-Ålesund, Svalbard (38, 39). The characteristics of subsurface active layer soil (from 18 to 27 cm depth) near the Bayelva Permafrost Monitoring Observatory are shown in Table S1. Soil moisture was 10.3%, soil organic matter was 2.5% dry weight, and the soil contained 10^6^ cells per g of wet soil and 45 ng DNA per g of wet soil. The concentrations of selected trace elements are shown in Table S1. Fe was abundant (2.3 × 10^5^ mg kg^−1^), while other biologically relevant trace metals such as Co, Ni, Cu, and Zn were present at <50 mg kg^−1^. Mg was present at 4,580 mg kg^−1^, and Mn was present at 478 mg kg^−1^.

### Microbial respiration upon thaw

CO_2_ concentrations in the microcosms increased within days following thaw and incubation at 4°C and continued to increase during the first 28 days (22.0 mmol g^−1^ day^−1^) (Fig. S3A). This indicates that soil microorganisms rapidly resumed activity following thaw, and that Arctic soil carbon budgets and greenhouse gas fluxes may be highly sensitive to episodic winter warming events with sustained positive-degree temperatures (65). We reason that the increased availability of liquid water (from thawing of soil ice to soil water, as well as added H_2_^16^O or H_2_^18^O) was the primary trigger for the rapid onset of microbial respiration and CO_2_ release following soil thaw (as discussed by Nikrad et al. [9]). CO_2_ concentrations in the headspace of incubation vials continued to increase beyond 28 days in two of five replicates (15.2 nmol g^−1^ day^−1^) and decreased or plateaued after 28 days in three replicates, suggesting that community-level growth ceased or slowed after 28 days in these microcosms (Fig. S3A).

Methane concentrations remained low and stable (near 2 nmol g^−1^ of soil) during the incubation (Fig. S3C), suggesting either (i) no substantial methane production or consumption in the thawed soil or (ii) a steady-state level of methane production and consumption. The headspace of our microcosms remained oxic throughout the incubation (Fig. S3B), which may have inhibited methanogenesis. We also carried out parallel anoxic incubations of active layer soil, which did not produce substantial increases in methane concentrations either (Fig. S4B). The minimal production of methane under anoxic headspace is consistent with prior studies of thawed Svalbard soils and could be attributed to low organic matter content of the soil (1.38% by weight) and also relatively high iron concentrations in the soil (Table S1) which promotes iron reduction in anoxic conditions and can suppress methanogenesis (24, 66).

### “Early” and “late” communities

**Fig 2:**
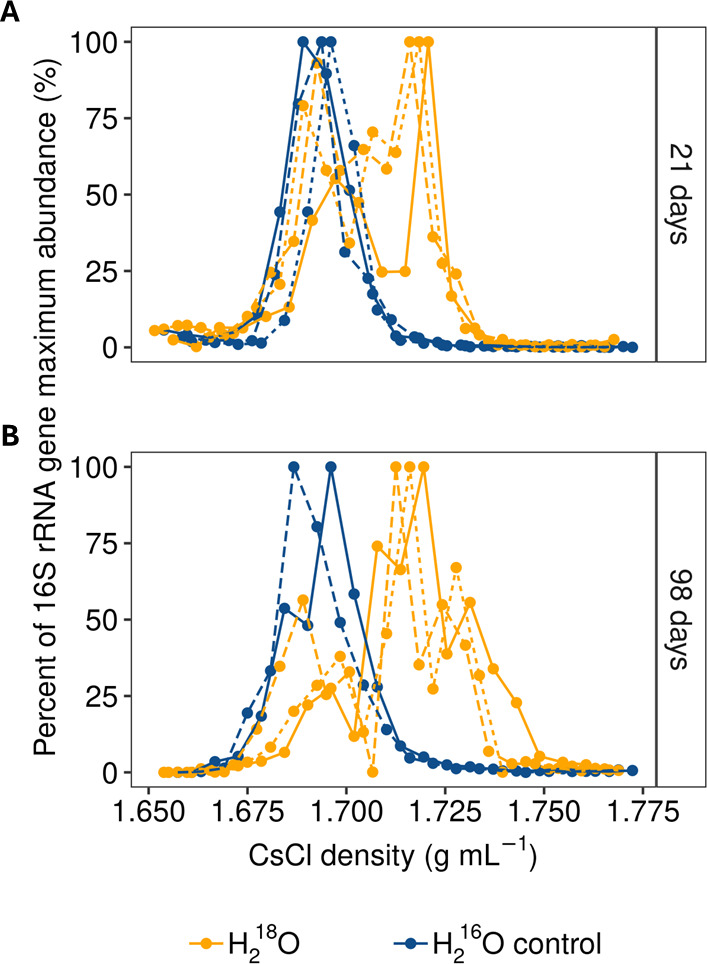
Density of DNA extracted from H_2_^18^O-amended and H_2_^16^O control microcosms. The abundances of 16S rRNA genes at different buoyant densities measured with qPCR at (**A**) 21 and (**B**) 98 days in oxic conditions are shown. Blue lines show the abundance of 16S rRNA genes in DNA extracted from the control microcosms that received H_2_^16^O water (i.e., H_2_^16^O control). The yellow lines show the abundance of 16S rRNA genes in DNA extracted from the microcosms that received H_2_^18^O water. The solid and dashed lines correspond to technical replicates. The *y*-axis values represent the percent of the 16S rRNA gene abundances normalized to the maximum value within the same replicate.

We detected microbial growth in the microcosms that were terminated after 21 days of incubation (Fig. 2). DNA extracted from soils that were incubated with H_2_^18^O water was enriched in ^18^O (hereafter “^18^O-labeled” DNA) relative to DNA extracted from soils incubated with H_2_^16^O (Fig. 2). At 21 days, a substantial portion (~30 to 45%) of the DNA in H_2_^18^O-amended microcosms remained unlabeled, indicating that a portion of the community did not grow and presumably remained dormant during the initial incubation period (Fig. 2A). We observed a shift to a heavier density range at the end of the incubation (i.e., at 98 days; Fig. 2B, yellow lines), indicating that microbial growth continued between 21 and 98 days. Despite continued growth from 21 to 98 days (Fig. S5B), 16S rRNA gene copy number did not increase during the incubation (Fig. S6C), suggesting that either early-growing cells replaced and destroyed dead or dormant cells or that some previously dormant cells started growing after 21 days.

**Fig 3:**
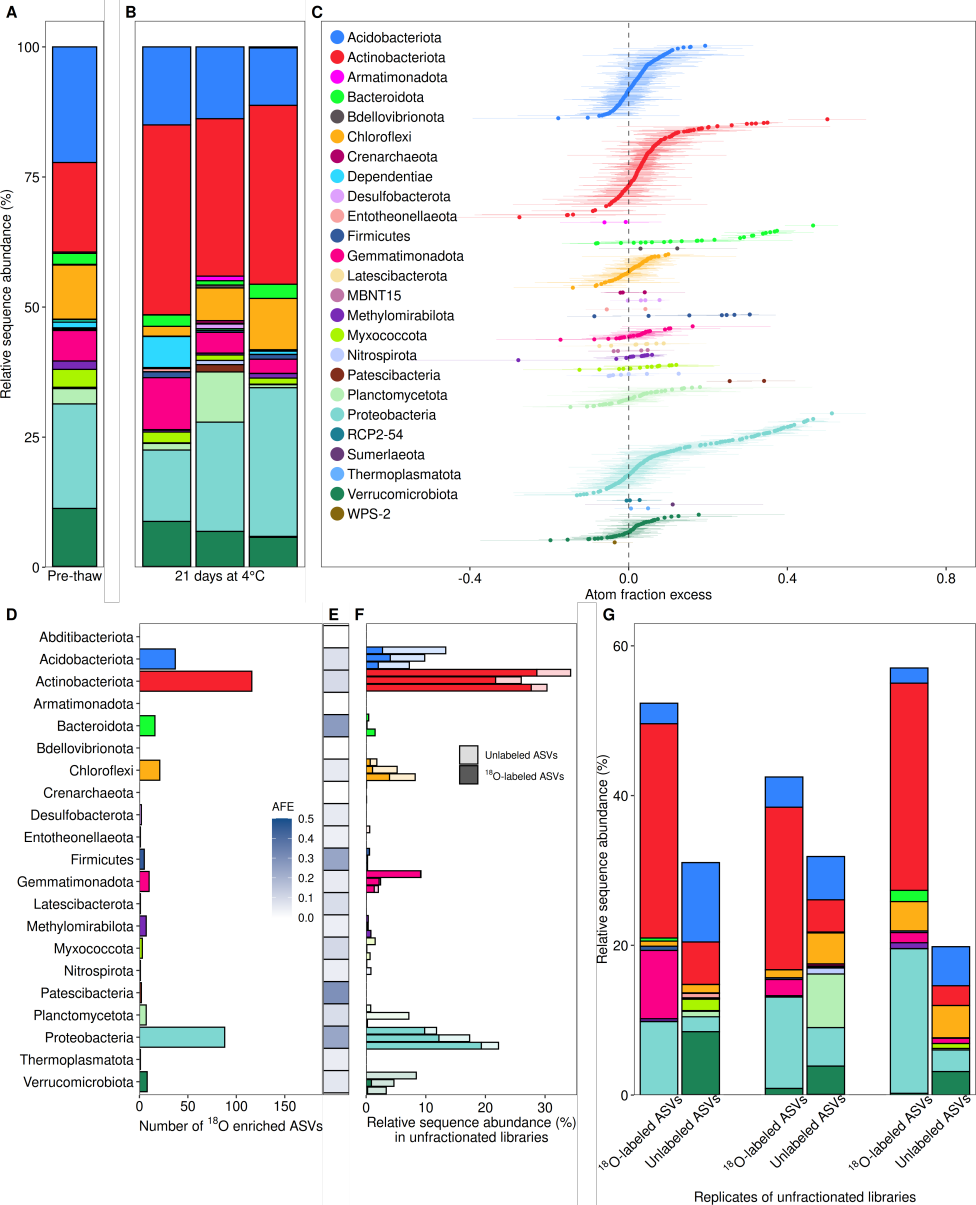
The composition of the ^18^O-labeled and unlabeled microbial community members after 21 days of incubation at 4°C under oxic conditions. (**A**) Relative 16S rRNA gene sequence abundance of phyla in frozen soil prior to incubation and thaw. (**B**) Relative 16S rRNA gene sequence abundance of phyla in soil after 21 days of incubation at 4°C. Bars show the community composition of triplicate microcosms. (**C**) AFE of ^18^O in DNA of ASVs. ASVs detected by qSIP (represented by colored dots) are grouped and colored by phylum and ordered along the *y*-axis by increasing AFE. Each dot represents the median AFE values, and error bars indicate the 90% confidence intervals. The further an ASV “dot” is to the right, the more enriched that ASV’s DNA is in ^18^O. AFE shows the magnitude of ^18^O incorporation into the DNA of growing microorganisms. (**D**) The number of ASVs that are enriched in ^18^O at 21 days. (**E**) Mean AFE values of ^18^O-labeled ASVs within each phylum. (**F**) The relative sequence abundance of ^18^O-labeled (darkly shaded) and unlabeled (lightly shaded) ASVs of each phylum. Only phyla to which at least one ^18^O-labeled ASV belongs (at 21 or 98 days) are shown. Bars show the abundance in triplicate microcosms. (**G**) The relative 16S rRNA gene sequence abundance of ^18^O-labeled and unlabeled ASVs in the unfractionated libraries of replicate microcosms. Seventeen to 26% of the relative sequence abundance of the unfractionated libraries was not detected in the fractionated 21 day qSIP libraries. We attribute this “not detected” ASV abundance to community differences between biological replicates, and it is inherent in the qSIP method, which relies on comparison between biological replicates to determine AFE. The ^18^O incorporation status of ASVs of this “not detected” fraction cannot be determined, and so the relative abundances of these are not shown in the plot.

**Fig 4:**
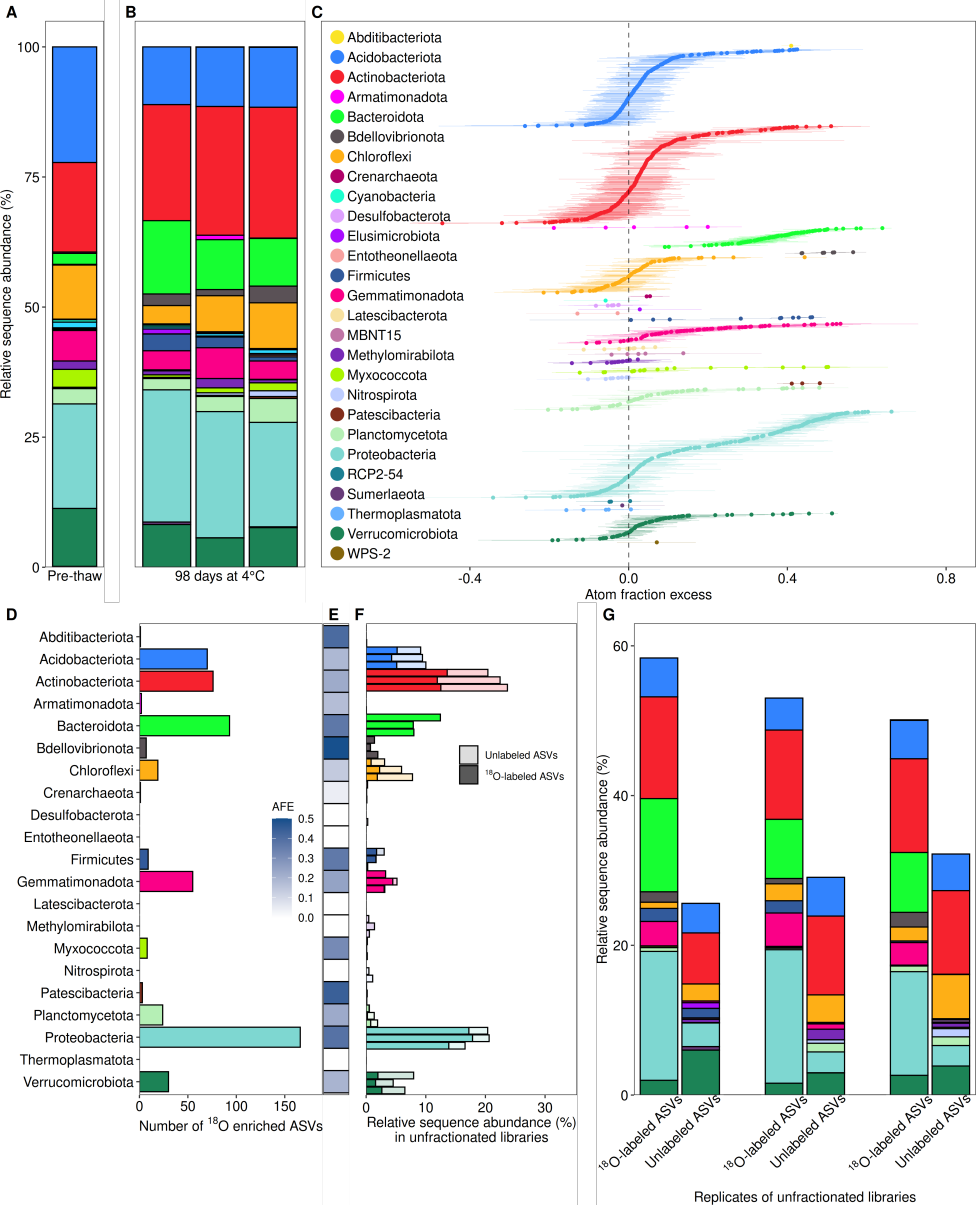
The composition of the ^18^O-labeled and unlabeled microbial community members after 98 days of incubation at 4°C under oxic conditions. (**A**) Relative 16S rRNA gene sequence abundance of phyla in frozen soil prior to incubation and thaw. (**B**) Relative 16S rRNA gene sequence abundance of phyla in soil after 98 days of incubation at 4°C. Bars show the community composition of triplicate microcosms. (**C**) AFE of ^18^O in DNA of ASVs. ASVs detected by qSIP (represented by colored dots) are grouped and colored by phylum and ordered along the *y*-axis by increasing AFE. Each dot represents the median AFE values, and error bars indicate the 90% confidence intervals. The further an ASV “dot” is to the right, the more enriched that ASV’s DNA is in ^18^O. AFE shows the magnitude of ^18^O incorporation into the DNA of growing microorganisms. (**D**) The number of ASVs that are enriched in ^18^O at 98 days. (**E**) Mean AFE values of ^18^O-labeled ASVs within each phylum. (**F**) The relative sequence abundance of ^18^O-labeled (darkly shaded) and unlabeled (lightly shaded) ASVs of each phylum. Only phyla to which at least one ^18^O-labeled ASV belongs (at 21 or 98 days) are shown. Bars show the abundance in triplicate microcosms. (**G**) The relative 16S rRNA gene sequence abundance of ^18^O-labeled and unlabeled ASVs in the unfractionated libraries of replicate microcosms. Sixteen to 18% of the relative sequence abundance of the unfractionated libraries was not detected in the fractionated 98 day qSIP libraries. We attribute this “not detected” ASV abundance to community differences between biological replicates, and it is inherent in the qSIP method, which relies on comparison between biological replicates to determine AFE. The ^18^O incorporation status of ASVs of this “not detected” fraction cannot be determined, and so the relative abundances of these are not shown in the plot.

The overall composition of the total microbial communities present at the “early” and “late” incubation time points was similar. ASV richness and evenness (as determined by Chao1 and inverse Simpson indices) and total abundance (as determined by qPCR of 16S rRNA genes) did not significantly change over the 98 day incubation (Fig. S6A through C), and at a phylum level, the composition of the initial (i.e., pre-thaw), 21 day, and 98 day microbial communities was generally similar (Fig. 3A and B and 4A and B) and did not differ statistically (community composition based on Bray-Curtis Dissimilarity Index of the 21 and 98 day microbial communities, ANOSIM, *R* = 0.15, *P* = 0.5).

### Taxon-specific growth following thaw

Microorganisms grew in thawed soils during the first 21 days and continued to grow during the 21 to 98 day incubation period (Fig. 2; Fig. S5). We observed taxon-specific differences in the growing portion of the 21 and 98 day communities which were not indicated in diversity index and community compositional comparisons of the total communities. In general, the microbial groups that were most relatively abundant at 21 and 98 days were also those that were labeled with ^18^O and therefore detected as growing (Fig. 3 and 4). Sequences attributed to Acidobacteriota, Actinobacteriota, and Proteobacteria collectively comprised 68% ± 3% (21 days) and 59% ± 1% (98 days) of the relative sequence abundance (Fig. 3B and 4B). These phyla also comprised 59% and 55% of the growing ASVs at 21 and 98 days, respectively (Fig. 3C and D and 4C and D). Acidobacteriota, Actinobacteriota, and Proteobacteria are commonly found to be dominant in permafrost-affected soils (24, 25, 35, 67). We also found that many of the ASVs belonging to these dominant groups did not grow, even after 98 days, indicating variable growth and dormancy responses between and within phyla after thaw.

While the overall composition of the total active layer “early” (21 day) and “late” (98 day) communities was similar, the growing members at 21 and 98 days were distinct from each other. Only 22% of ASVs that were ^18^O-labeled at 21 or 98 days were ^18^O-labeled at both time points. Observed richness (measured by unique ASV abundance) of ^18^O-labeled Acidobacteriota, Bacteroidota, Firmicutes, Gemmatimonadota, Myxococcota, Planctomycetota, Proteobacteria, and Verrucomicrobiota increased from the “early” to “late” communities (Fig. 3D and 4D), suggesting that the factors that triggered and supported the growth of members of these phyla persisted or were enhanced during the later stages of the incubation. This is in agreement with previous work showing increases in the relative abundance of Bacteroidota and Verrucomicrobiota species between the beginning (June) and end (October) of the thaw season in soils surrounding Ny-Ålesund, Svalbard (68). Gemmatimonadota, Planctomycetota, and Verrucomicrobiota are generally considered to be “slow growing” (i.e., with doubling times of days to weeks) (69, 70). The ability of a microbial cell to rapidly resume metabolic activity following dormancy, as indicated here by CO_2_ production within days of soil thawing, may suit organisms in habitats that frequently experience fluctuating environmental conditions (71–73). Slow growth is also favored by organisms residing in harsh environments (74–76). ASVs affiliated with Bdellovibrionota, Abditibacteriota, Armatimonadota, and Crenarchaeota were only ^18^O-labeled in the “late” communities (and not in the “early” communities) and thus only grew after a longer period of thaw, indicating that the duration of thaw shapes the growth response of the soil microbial community.

Dominant groups in the “early”- and “late”-growing communities may be influenced by nutrient availability and turnover. Actinobacteriota, Bacteroidota, and Proteobacteria, which are prominent in the “early”-growing community, are associated with efficient necromass recycling (77) and may benefit from necromass released from frozen soil upon thaw (78). Moreover, groups broadly associated with the degradation of complex and specific carbon sources were more prominent in the “late”-growing community, including the Armatimonadota, Verrucomicrobiota, and Planctomycetota (79). These groups may be more competitive following the exhaustion of labile carbon sources by “early” growers (80). The disappearance of “early” ^18^O-labeled ASVs by 98 days (Methylomirabilota, Thermoplasmotota, Entotheonullaeota, Latescibacterota, Nitrospirota, and Desulfobacterota) could be attributed to the turnover of these groups, potentially due to predation (see “Epibiont and predatory phyla”) or cell death and subsequent degradation of the cellular DNA.

Bacteroidota, despite their low relative 16S rRNA gene abundance (0.8%–2.7% and 9.1%–14.0% at 21 and 98 days, respectively), were disproportionately ^18^O-labeled, and all ASVs affiliated with Bacteroidota were ^18^O-labeled at 98 days (Fig. 4C). Bacteroidota ASVs grew during the “early” stage, and their growth was sustained in the “late” stage, with AFE of this group increasing by 0.16 between 21 and 98 days (Fig. S7). Some of these ASVs were the most isotopically enriched ASVs in the entire soil community (Fig. 3C and 4C). Overall, this suggests that the growth of Bacteroidota may play a predominant role in post-thaw microbial dynamics despite their relatively low abundance. Bacteroidota are widespread in soil (81) including in Svalbard active layer soils (26). They are competitive degraders of complex carbohydrates owing to efficient and substrate-specific enzyme production and a phylum-specific secretion system that provides gliding motility used for substrate foraging and tethering of enzymes for concentrating substrate-degrading activity near the cell (82).

### Epibiont and predatory phyla

Members of phyla often associated with predation in soil, including Myxococcota, Patescibacteria, and Bdellovibrionota, were ^18^O-labeled following thaw (Fig. 3C and 4C). Myxococcota prey on other bacterial cells (83) and have been implicated as key bacterial predators in permafrost-affected environments (34, 84), owing to their broad host range (85). Patescibacteria (all belonging to the class Saccharimonadia) and Bdellovibrionota (including Oligoflexia spp., *Bacteroivorax* sp., *Bdellovibrio* sp., and *Silvanigrella* sp.) also grew following thaw (Fig. 3C and 4C). Saccharimonadia are obligate epibionts, growing while attached to surfaces of hosts (86, 87), and show host specificity to members of the Bacteroidota and Actinobacteriota (87, 88). Bdellovibrionota are also known predatory bacteria (89–92). Despite making up <1% of the total relative sequence abundance (Fig. 4B), all detected ASVs of Patescibacteria were ^18^O-labeled and growing. Similarly, all detected ASVs of Bdellovibrionota, making up just 2 ± 1% of relative sequence abundance, were ^18^O-labeled after 98 days. These phyla, while low in relative abundance, were the most ^18^O-labeled groups by 98 days, potentially as a result of the consumption of ^18^O-enriched hosts and prey, as well as ^18^O-labeled water incorporation during growth. Observations indicate that obligately predatory bacteria are >60% more active than their non-predatory counterparts when new carbon sources become available (93), as is likely to occur when soil thaws, which may contribute to the high AFE of predatory phyla observed here. Notably, the AFE of ASVs affiliated to Bdellovibrionota and Patescibacteria present at both 21 and 98 days increased by a mean value of 0.46 and 0.16, respectively (Fig. S7), suggesting continued growth and predation or epibiosis for ASVs belonging to these phyla during sustained thaw. In contrast, ASVs affiliated to Myxococcota decreased in AFE on average over this time, suggesting that turnover may differ between different predatory groups, potentially depending on the growth cycles of prey or host bacteria. While the Myxococcota host range is broad, the Bdellovibrionota host range is narrower (85), and Bdellovibrionota growth may depend more strongly upon prior growth of their host species, contributing to their later ^18^O-labeling and growth seen here. The growth dynamics of prey species may additionally affect the timing of predatory activity. Specifically, prey can use dormancy and slow growth as predator-avoidance strategies (94–97).

### Methanotrophic organisms

**Fig 5:**
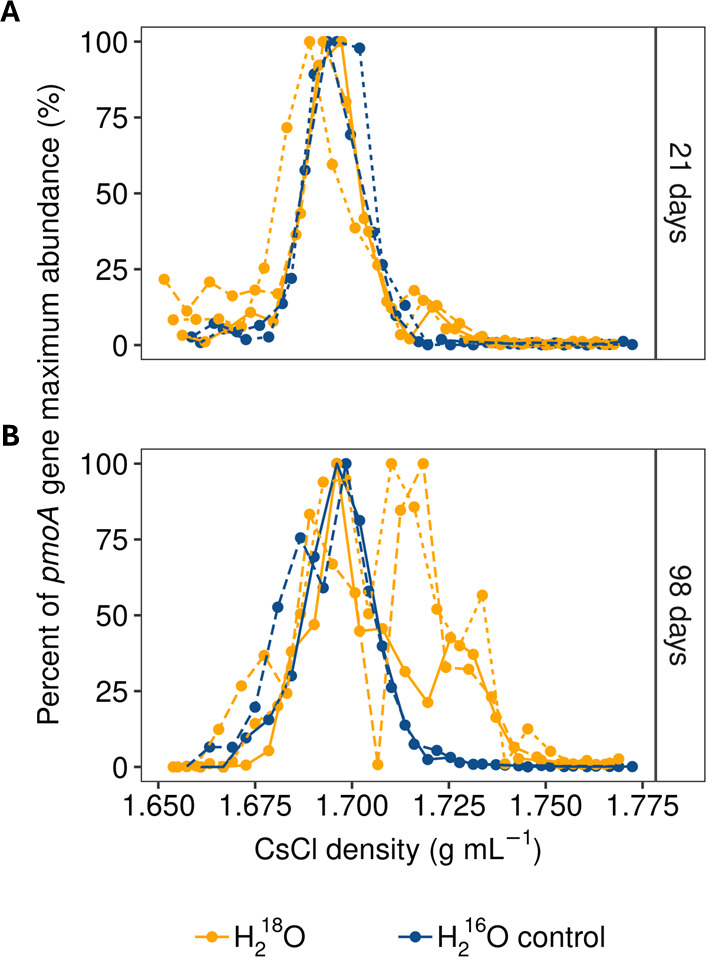
Density of *pmoA* genes extracted from H_2_^18^O-amended and H_2_^16^O control microcosms. The abundances of *pmoA* genes at different buoyant densities measured with qPCR at (**A**) 21 and (**B**) 98 days of oxic incubation at 4°C are shown. Blue lines show the abundance of *pmoA* genes in DNA extracted from the microcosms that received H_2_^16^O water (i.e., H_2_^16^O control). The yellow lines show the abundance of *pmoA* genes in DNA extracted from the microcosms that received H_2_^18^O water. The solid and dashed lines correspond to technical replicates. The *y*-axis values represent the percent of the *pmoA* gene abundances normalized to the maximum value within the same replicate.

Methane concentrations remained low during the incubations (Fig. S3C), and we did not detect any 16S rRNA genes that could be attributed to members of the methanogen-affiliated Euryarchaeota or Halobacterota, or any *mcrA* genes indicative of methanogenesis biosynthetic pathways. We measured an isolated increase in methane in a single measurement from a single microcosm (Fig. S3C). This isolated increase was not consistent with the overall trend of methane concentrations, nor did it co-occur with a drop in oxygen concentrations or a detectable methanogen population in the 16S rRNA gene amplicon libraries. Thus, we have cautiously disregarded it. Despite the absence of obvious methane production or consumption, methanotrophs affiliated to *Methylocapsa* of the family *Beijerinckiaceae* and an unknown member of the *Methyloligellaceae* were ^18^O-labeled during the 21 day incubation. We also saw some evidence of the growth of aerobic methane oxidizers during the 98 day incubations, with notable labeling of the *pmoA* gene (Fig. 5B) which encodes the particulate methane monooxygenase enzyme that facilitates the conversion of methane to methanol during aerobic methane oxidation (98). The aggregate ^18^O-labeled *pmoA* gene DNA (yellow density peak ~1.710 to ~1.730 g mL^−1^, Fig. 5B) was of similar abundance to the aggregate non-labeled *pmoA* gene DNA (yellow density peak ~1.690 g mL^−1^, Fig. 5B), indicating that up to half of the *pmoA*-containing cells grew during the 98 days following thaw.

No 16S rRNA gene amplicons affiliated with known methanotrophic clades were ^18^O-labeled at 98 days. Several members of the nitrite-reducing, methane-oxidizing Methylomirabilota were ^18^O-labeled after 21 days; however, all were affiliated with the non-methanotrophic Rokubacteriales, among which the *pmoA* gene has not been detected (99). None of the ^18^O-labeled ASVs affiliated with Verrucomicrobiota were affiliated with the methanotrophic clades of this phylum, which are *Methylacidiphilum* and *Methylacidimicrobium* (100). It is possible that growing methanotrophs (as detected by labeled *pmoA*-containing organisms) in 98 day incubations belonged to a clade or clades for which the methanotroph capability is not yet known. The PCR primers we used in this study are highly specific to the *pmoA* gene, unable to amplify closely related sequences such as *amoA*, the gene for ammonia oxidation (101). These primers will not amplify divergent *pmoA*-like genes such as those found in methane-oxidizing members of the Verrucomicrobiota and Methylomirabilota (102) or from novel taxa, and we therefore expect to have underestimated the true concentration of *pmoA*-like genes affiliated with methane oxidation. Many *pmoA*-containing microbial lineages have no cultured representatives (98, 103). The presence of ^18^O-labeled *pmoA* genes may alternatively suggest the growth of facultative methanotrophs that are able to grow on other carbon sources when methane is limiting, such as members of the *Methylocapsa* or the largely uncultured USCα group, which can use other C1 compounds such as methanol or acetate when methane is not readily available (104, 105). Methanotroph diversity is low in thawed permafrost-affected soils (106, 107), so the ^18^O-labeled *pmoA* DNA in our incubations might have derived from a low number of uncultivated *pmoA*-containing species. Methylotrophs known to utilize methanol (a by-product of methane oxidation) and other C1 compounds were ^18^O-labeled during the 21 and 98 day incubations. These included *Bosea* sp. of the family *Beijerinckiaceae*, *Hyphomicrobium* sp. of the family *Hyphomicrobiaceae*, and *Methylotenera* sp. of the family *Methylophilaceae* (108).

### Microbial dormancy

Soils are regarded to harbor a diverse, yet low-abundance, dormant microbial community (109). In our microcosms, approximately half of the ASVs in thawed active layer soils remained unlabeled. These non-growing ASVs contributed 27% ± 6% (21 days) and 29% ± 3% (98 days) of the relative 16S rRNA gene sequence abundance. These non-growing ASVs were diverse and affiliated with many phyla. No ASVs associated with the phyla Cyanobacteria, Elusimicrobiota, Entotheonellaeota, MBNT15, RCP2-54, Sumerlaeota, or WPS-2 were ^18^O-labeled at 21 or 98 days. Thus, despite these groups being present in the total community, we did not detect any growing organisms from these clades. The relative 16S rRNA gene sequence abundances of these same non-growing ASVs were abundant in parallel anoxic microcosms (30% ± 9% at 21 days and 35% ± 3% at 98 days). We therefore suggest that the reason these ASVs did not grow during oxic thaw at 4°C is a combination of different requirements specific to each clade and not simply because they required anoxic conditions for growth. These non-growing ASVs may require additional nutrients or other environmental characteristics not provided to them by the soil, their immediate environment, or the conditions of the microcosm in order to grow. For example, certain microorganisms depend on the availability of biologically active trace elements (biometals, as defined by Giovannelli [110]) as cofactors for key biogeochemical reactions important in organic carbon degradation and overall carbon cycling (111). Physico-chemical factors including pH and the availability of key trace elements might restrain growth of certain groups or increase resource competition. Additionally, some microorganisms require trace quantities of the atmospheric gases H_2_ or CO (112) which may not have been sufficiently available to them in the microcosm headspace.

Overall, this work highlights that cell dormancy, or very slow growth, contributes to the total microbial diversity of this subsurface active layer Arctic soil during thawing. Consideration of this dormant or slow-growing fraction in soil microbial-biogeochemical models will improve the mechanistic realism and predictions of carbon dynamics and emissions from soil on seasonal timescales (30, 113, 114) and is necessary for understanding biogeochemical transformations in dynamic environments such as seasonally variable Arctic systems (31). Should nutrients or environmental factors be encountered that promote the growth of dormant cells, rapid recruitment from this dormant population to the growing population may occur (72, 109). The physical connectivity across and throughout active layer soils potentially contributes to the transportation and dispersal of both nutrients and dormant cells (115).

Additionally, some of the unlabeled ASVs in this study may be dead (i.e., extracellular DNA (116) or necromass) or growing very slowly, such that growth cannot be detected over 98 days using qSIP. However, the impact of extracellular DNA on microbial community composition after 21 and 98 days of incubation is likely to be small owing to degradation of extracellular DNA associated with microbial necromass consumption and replacement of extracellular DNA with DNA from recently growing clades (117, 118). Overall, we found that active layer soils harbored both microorganisms that are primed to grow after soil thaw and others that form a seed bank and remain dormant, dead, or in a very slow mode of growth.

### Seasonality of Arctic soils and the impact of climate warming

About 28% of permafrost area in northern regions is likely to experience thaw if global warming stabilizes at 2°C above pre-industrial levels (119). Meanwhile, the mean thickness of the active layer in the northern polar region has increased by 0.11 cm per year between 2003 and 2020 due to thawing of underlying permafrost (6). Even under optimistic emissions scenarios, the global active layer soil volume is projected to significantly increase as Arctic permafrost thaws (13), causing a substantial amount of permafrost carbon to undergo seasonal thawing. The warming Arctic climate is also likely to cause an increase in the number of days per year that active layer soils experience temperatures above 0°C (10) (Fig. S8). We anticipate that, with the lengthening of the thawed period, the activity of “late”-growing microorganisms (here represented by those growing after 98 days of thaw) will become increasingly influential on carbon transformations and greenhouse gas emissions from permafrost-affected regions. The increase in taxonomic richness that we observed in the “late”-growing community may also equip soils with richer biogeochemical functions and metabolisms. For example, we found that the abundance of ^18^O-labeled *pmoA* genes from growing microorganisms was higher in the “late”-growing community than the “early” communities (Fig. 5). Notably, we did not observe a corresponding increase in methane concentrations in our microcosms, suggesting that the “late”-growing community detected here may have oxidized any methane released from the thawed soil. Similarly, the lower degree of ^18^O-labeling in *pmoA*-bearing microorganisms in the “early” community suggests that this community may be, compared to the “late”-growing community, relatively less prepared to oxidize trapped methane that is released following thawing of active layer or permafrost soils. The presence of growing microorganisms capable of methane oxidation is consistent with recent observations of net negative methane fluxes from the soil to the atmosphere (i.e., methane uptake) in high Arctic tundra during the summer season in Svalbard (120) and northern Canada (121, 122).

### Changing hydrology

Our microcosm-based study did not account for dynamic hydrological regimes that may occur during natural seasonal thawing. Hydrological changes, including the inundation and draining of soil, may alter redox status and replenish carbon substrates and other nutrients, altering or enhancing rates of microbial activity, growth, and biogeochemical rates (123). For instance, methane oxidation may be enhanced in the drier portion of the summer in increasingly aerated soil (120). At our study site, observations show that soil moisture at 22 cm below the soil surface rises from ~8% (volume) to ~32% after thaw (44), potentially affecting microbial community structure and growth (124–127). In our microcosm experiments, the addition of H_2_^18^O water to create slurries increased the liquid water content by ~5 times, potentially altering the post-thaw microbial community structure relative to *in situ* active layer soil. Nevertheless, our findings closely mirror *in situ* observations of the dominant phyla in active layer soil (68), supporting the relevance of our microcosm study to understanding active layer soil dynamics.

### Conclusions

High Arctic regions, including Svalbard, are among the fastest-warming places on Earth (36, 37). This warming is resulting in permafrost degradation and longer active layer thaw seasons. We used DNA qSIP with H_2_^18^O (33) to trace taxon-specific growth following active layer thawing for 21 and 98 days. We revealed time-dependent microbial community dynamics, including distinct “early” and “late” stage growers, a notable prevalence of dormancy, and potential microbial predation, death, and turnover. Notable CO_2_ production (attributed to the resumption of microbial activity) occurred within days of thawing, indicating that Arctic soil carbon is susceptible to microbial degradation during short-lived winter warming events when soil temperatures surpass 0°C (65). After 21 and 98 day incubations, approximately half of the diversity in active layer soil was attributed to microorganisms that did not measurably grow, underscoring the prevalence of dormancy even during thawed summer-like conditions and the value of carrying out physiology-based microbial assays for understanding microbial community dynamics in thawing Arctic soils. We found that bacterial predation and epibiosis are important factors shaping microbial community dynamics in seasonally thawed Arctic soils. We did not observe methane production or consumption, yet we detected a growing community that was primed for methane oxidation after thaw. Our study reveals that Arctic soils harbor a microbial community that undergoes complex growth, predation, and dormancy dynamics during seasonal thawing—processes that shape the seasonal population structure and regulate carbon storage dynamics. These high Arctic soil habitats are exceptionally vulnerable to warming and contemporary climate change, leading to further alterations of soil ecological dynamics and carbon cycling.

## Supplementary Material

Reviewer comments

## Data Availability

DNA sequence data can be found in the NCBI Sequence Read Archive under the BioProject accession PRJNA1118335. Soil core and pit physical and geochemical data can be found in the Zenodo archive at https://doi.org/10.5281/zenodo.19084687.

## SUPPLEMENTAL MATERIAL

Supplemental Information msystems.00738-25-s0001.docx Supplemental text, figures, and tables.

OPEN PEER REVIEW reviewer-comments.pdf An accounting of the reviewer comments and feedback.

ASM does not own the copyrights to Supplemental Material that may be linked to, or accessed through, an article. The authors have granted ASM a non-exclusive, world-wide license to publish the Supplemental Material files. Please contact the corresponding author directly for reuse.
