# Supplementary material for: Seasonal thawing of high Arctic soils triggers selective microbial growth and predation
Source: mSystems. 2026 May 7;11(6):e00738-25. doi: 10.1128/msystems.00738-25 (PMC13288927; doi:10.1128/msystems.00738-25)
Supplement: Supplemental Information — Supplemental text, figures, and tables. [file msystems.00738-25-s0001.docx]

Title: Seasonal thawing of high Arctic soils triggers selective microbial growth and predation

Authors: Margaret A. Cramm^1,2,3^, Ömer K. Coskun^4^, Francesco Montemagno^5^, Matteo Selci^5,6^, Daniel S. Read^7^, Tim Goodall^7^, Brianna Green^8^, Sayali A. Mulay^9,10^, Katie Sipes^9^, Andrey A. Abramov^11^, Catherine M. Heppell^12^, Julia Boike^13,14^, Donato Giovannelli^5, 6, 15, 16,17^, Tatiana A. Vishnivetskaya^9^, Robert L. Hettich^10^, Andrew D. Steen^8,9,18^, Karen G. Lloyd^9,19^, William D. Orsi^4,20^, Anne D. Jungblut^3^, James A. Bradley^2,21^

Affiliations:

^1^ School of Geography, Queen Mary University of London, London, UK

^2^ School of Biological and Behavioural Sciences, Queen Mary University of London, London, UK

^3^ Department of Science, Natural History Museum, London, UK

^4^ Department of Earth and Environmental Sciences, Paleontology & Geobiology, Ludwig-Maximilians-Universität München, Munich, Germany

^5^ Department of Biology, University of Naples Federico II, Naples, Italy

^6^ Department of Marine and Coastal Sciences, Rutgers University, New Brunswick, NJ, USA

^7^ UK Centre for Ecology & Hydrology, Wallingford, UK

^8^ Department of Earth and Planetary Sciences, University of Tennessee, Knoxville, TN, USA

^9^ Department of Microbiology, University of Tennessee, Knoxville, TN, USA

^10^ Biosciences Division, Oak Ridge National Laboratory, Oak Ridge, Tennessee, USA

^11^ Kovda Institute of Physicochemical and Biological Problems in Soil Science, Russian Academy of Sciences, Pushchino, Russia

^12^ Chilterns National Landscape, Chinnor, UK

^13^ Permafrost Research, Alfred Wegener Institute Helmholtz Centre for Polar and Marine Research, Potsdam, Germany

^14^ Geography Department, Humboldt- Universität of Berlin, Berlin, Germany

^15^ Institute for Marine Biological Resources and Biotechnologies, Italian National Research Council, CNR-IRBIM, Ancona, Italy

^16^ Earth-Life Science Institute, ELSI, Tokyo Institute of Technology, Tokyo, Japan

^17^ Marine Chemistry and Geochemistry Department, Woods Hole Oceanographic Institution, Woods Hole, MA, USA

^18^ Department of Biological Sciences, University of Southern California, Dornsife College of Letters, Arts and Sciences, Los Angeles, California, USA

^19^ Department of Earth Sciences, University of Southern California, Dornsife College of Letters, Arts and Sciences, Los Angeles, California, USA

^20^ GeoBio-Center LMU, Ludwig-Maximilians-Universität München, Munich, Germany

^21^ Aix Marseille Univ, Université de Toulon, CNRS, IRD, MIO, Marseille, France

Supplementary methods

*Soil moisture, organic matter content, trace elements, and cell abundance*

Change in weight before and after drying was used to determine soil moisture content. We weighed the soil before and after drying at 105 °C in a drying oven (OP100, LTE Scientific LTD.) overnight. We carried out sequential loss on ignition on the dried soil to measure the concentration of organic matter. We placed dried soil in a furnace (AAF 1100 Carbolite) at 550°C overnight (~16 hours). We measured the soil organic matter content using the change in dry weight before and after heating in the furnace.

We measured trace elements used as cofactors in biogeochemical cycles (Hay Mele et al. 2023) in triplicate from dried soils using acid assisted microwave digestion followed by Inductively Coupled Plasma Mass Spectrometry determination (Correggia et al. 2023). Briefly, we treated dried soils with concentrated HNO_3_ in a microwave reactor (Multiwave GO Plus, Anton Paar). We removed the acid-digested soils by centrifugation, and we analysed the supernatant, diluted 100- to 10,000-fold, using an inductively coupled plasma-mass spectrometer (ICP-MS, Agilent 7900) under helium collision mode applying an ultra high-matrix setting to reduce polyatomic interferences.

We carried out cell quantification in triplicate using flow cytometry at the UK Centre for Ecology & Hydrology according to Khalili et al. (2019). Briefly, we mixed 0.4 g soil with 18.75 mL detergent solution (50 mM tetrasodium phosphate pH8 with 0.5% Tween80) and 1.25 mL 4% Paraformaldehyde and incubated it at 4°C overnight. We shook the soil solution at 200 rpm for 30 min at room temperature in an orbital shaker. After settling for 1 min, we layered 1 mL of the upper phase slowly onto 0.5 mL Nycodenz cushion (80% w/v Nycodenz in 50mM tetrasodium phosphate, 0.2 µm filtered) and centrifuged at 14,000 × g for 30 minutes. We recovered the upper and middle phase (1 mL), mixed it with 1:1 with TSP (50 mM tetrasodium phosphate buffer) and centrifuged it at 17,000 × *g* for 25 minutes. We resuspended the pellet in 0.5 mL TSP, stained 0.15 mL of each sample with SYBR green I, and analysed it on a Thermo Fisher Attune CytPix recording green fluorescence channel (FL1) and forward scatter (FSC-H) with gating to count stained cells.

*DNA extraction*

To extract DNA from microcosm replicates (3) in which unfractionated, whole community libraries were created, 1 mL of filter- and UV-sterilized extraction buffer was added to 0.5 g slurry or soil in 2 mL Lysing Matrix E Tubes (MP Biomedicals) and homogenized at 6,500 rpm for 40 s in a Precellys 24 Lysis Homogeniser (Bertin). We heated the homogenized slurry at 98 °C for 5 min, followed by freezing, a second 98 °C heat treatment for 5 min, and followed by a second 40 s homogenization at 6,500 rpm. We centrifuged the lysate at 13,000 × *g* for 10 min. The supernatant was put into 50 MW KDa Amicon Ultra filters and centrifuged at 4,000 × *g* until it was concentrated to 100 µL.

DNA from the H_2_^18^O-amended replicate and a single H_2_^16^O control replicate, was fractionated according to its density. DNA from these microcosms was extracted from the entire microcosm slurry according to Coskun et al. (2018) as above with the following modifications. The microcosm slurry was added to 6 mL C1 extraction buffer in 15 mL Lysing Matrix E Tubes (MP Biomedicals). Homogenization occurred at 6.0 m s^-1^ for 40 s using a FastPrep-24 5G homogenizer (MP Biomedicals). We centrifuged the lysate at 4,700 rpm for 5 min. We then transferred the supernatant to 30 MW KDa Amicon Ultra filters and centrifuged it down to 100 µL. We purified the DNA using the Qiagen DNeasy PowerClean Pro Cleanup kit and quantified as above.

*16S rRNA gene amplification with PCR for library preparation and sequencing*

The PCR reaction mix (25 µL) was composed of 1 µL of DNA template, 2.5 µL of each primer (1 µM), 6.5 µL of PCR-grade water, and 12.5 µL KAPA 2G Fast HotStart ReadyMix (Roche). The 35-cycle protocol included an initial 95 °C for 3 min step followed by 10 cycles of denaturing at 95 °C for 30 s, annealing at temperatures dropping from 60 to 51 °C each for 45 s, and extension at 72 °C for 1 min. Following these were 25 similar cycles but with a consistent annealing temperature of 55 °C. The protocol ended with a 5 min extension at 72 °C.

Supplementary Figures


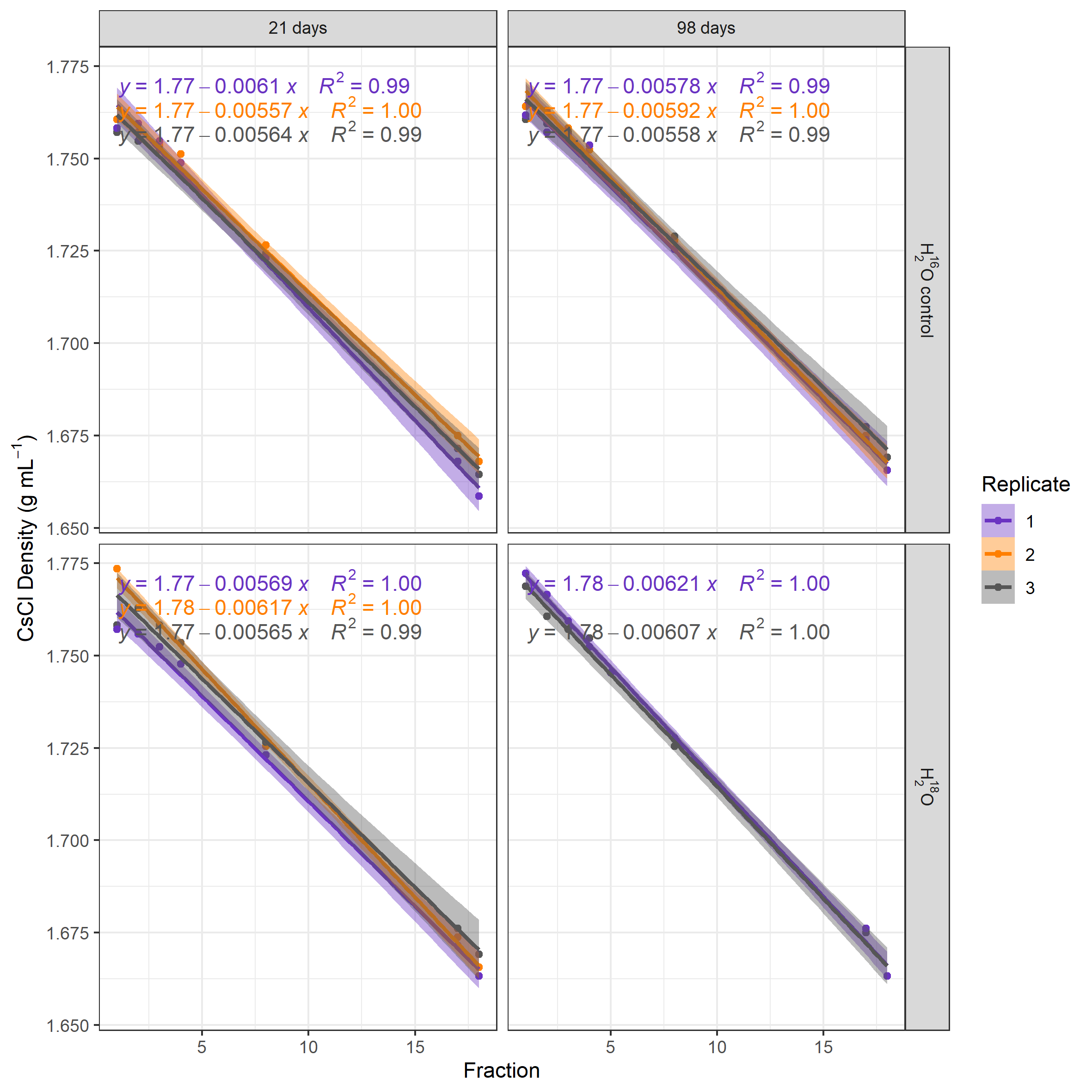


Fig. S1: Density of fractions of the CsCl density gradient. Lines show a linear model of the density of each fraction. The shaded area shows the 0.95 confidence interval.


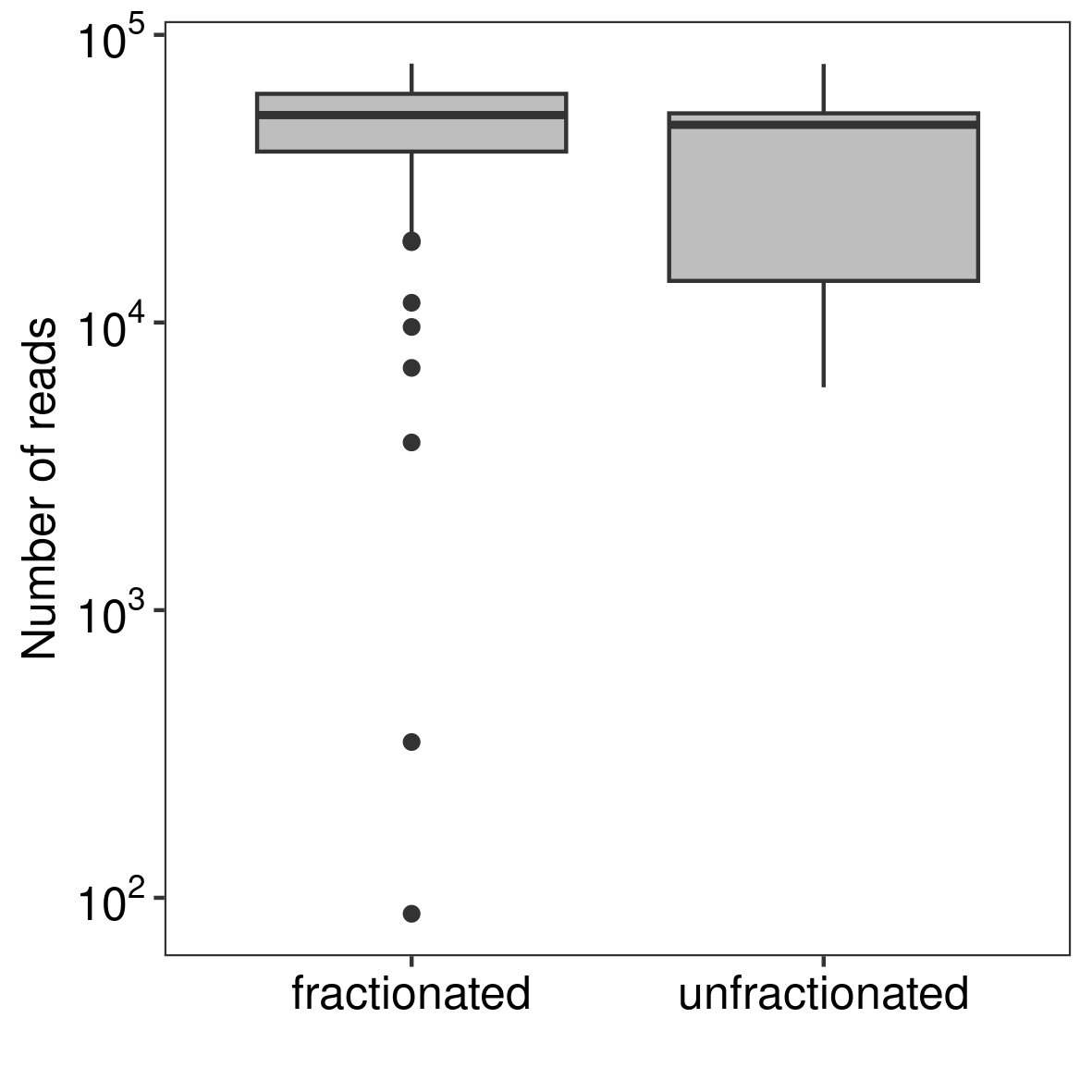


Fig. S2: Read abundance in non-rarefied sequencing libraries. The number of reads (log10) in the fractionated and unfractionated libraries after filtering. The median library size was 51,343 reads. The median library size was 52,604 for fractionated samples (n = 132), and 48,677 for unfractionated samples (n = 8).


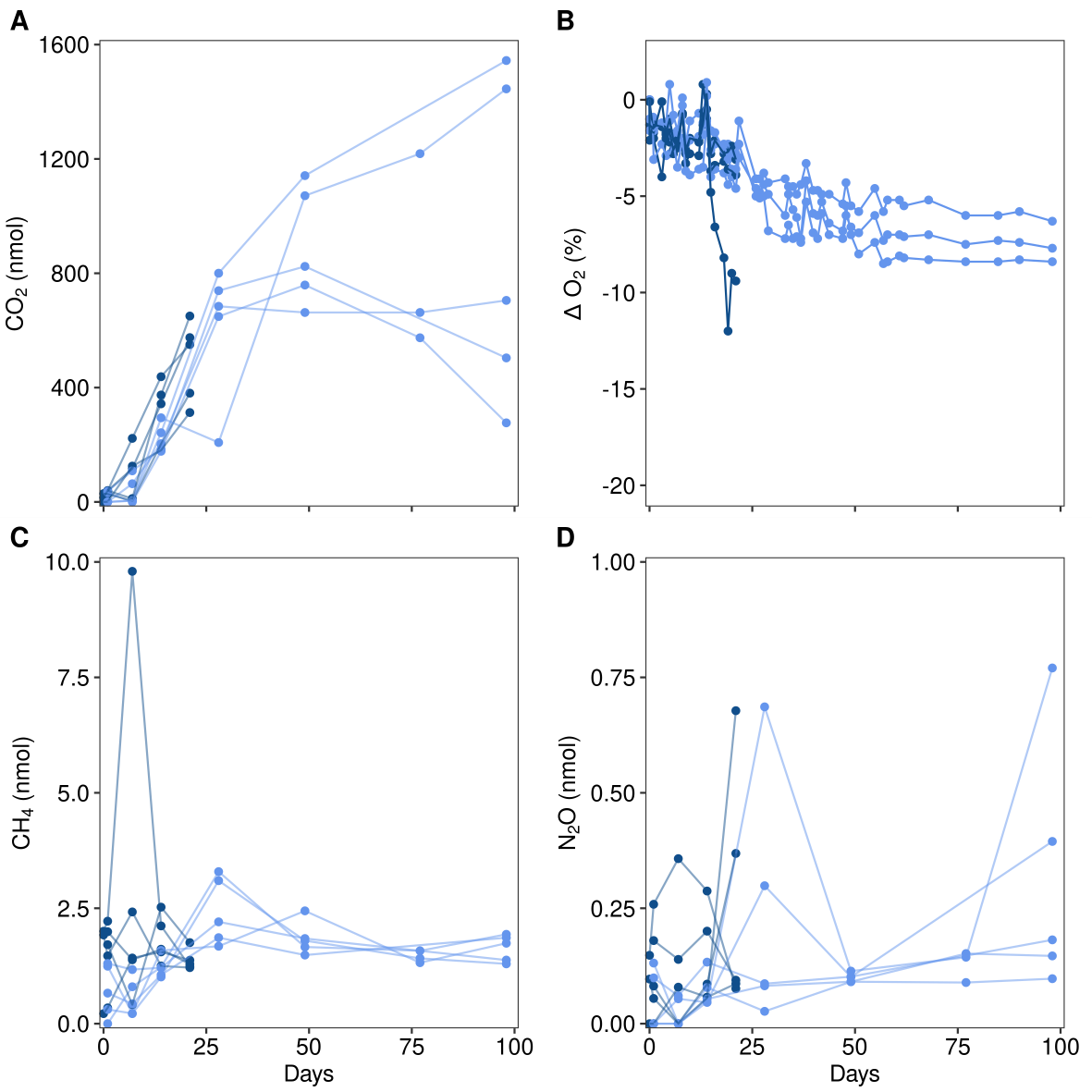


Fig. S3: Headspace gas during oxic 4 °C incubation. (A) CO_2_, (C) CH_4_, and (D) N_2_O abundance and (B) change in absolute O_2_ concentration (∆O%) in the headspace of microcosms (5 replicates) of subsurface active layer (18-27 cm depth) soil incubated over 21 days (dark blue) and 98 days (light blue) at 4°C under oxic conditions. Gas abundances for CO_2_, CH_4_, and N_2_O were normalized to per g of wet soil incubated.


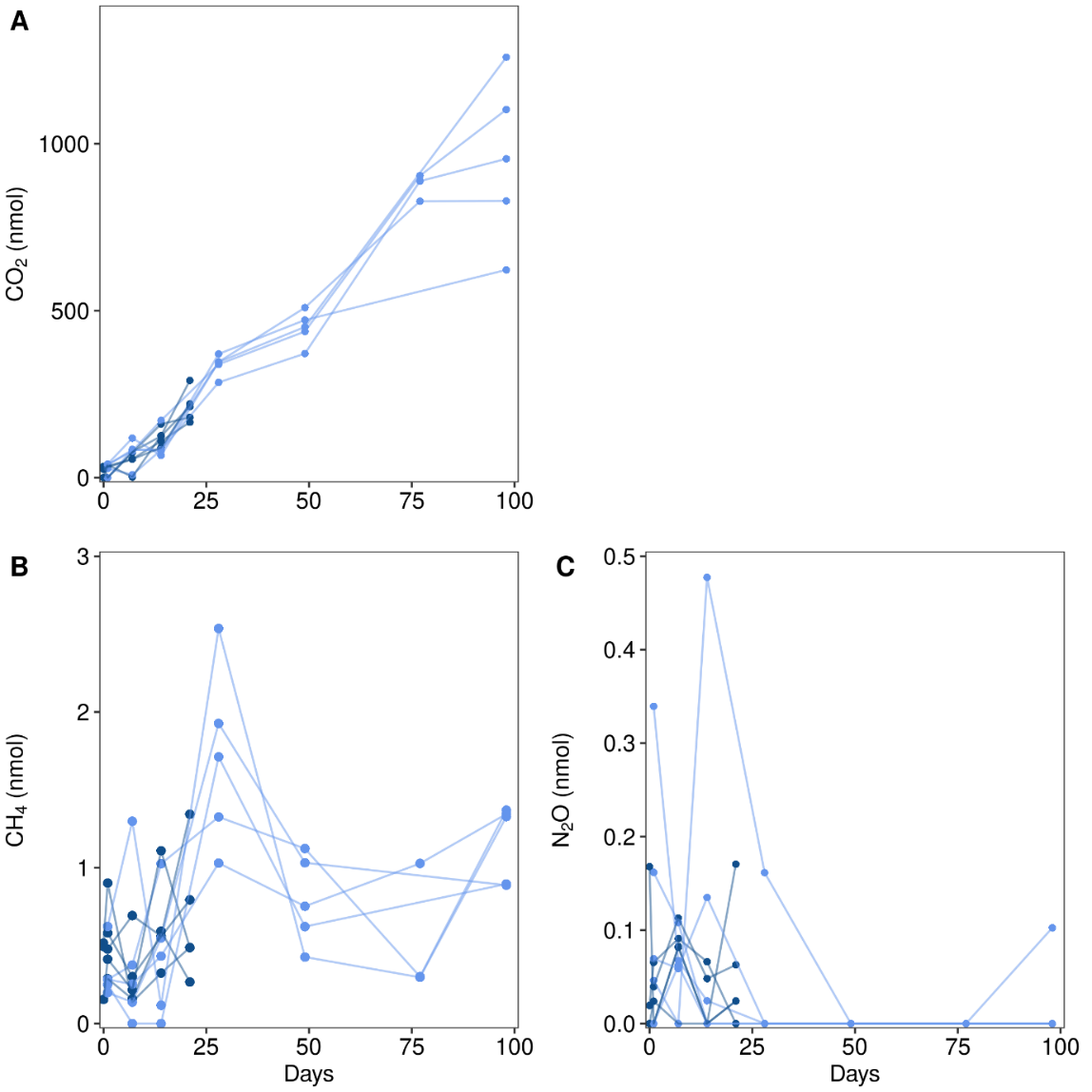


Fig. S4: Headspace gas during anoxic 4 °C incubation. (A) CO_2_, (B) CH_4_, and (C) N_2_O abundance in the headspace of microcosms (5 replicates) of subsurface active layer (18-27 cm depth) soil incubated over 21 days (dark blue) and 98 days (light blue) at 4°C under anoxic conditions. Gas abundances were normalized to per g of wet soil incubated.

**
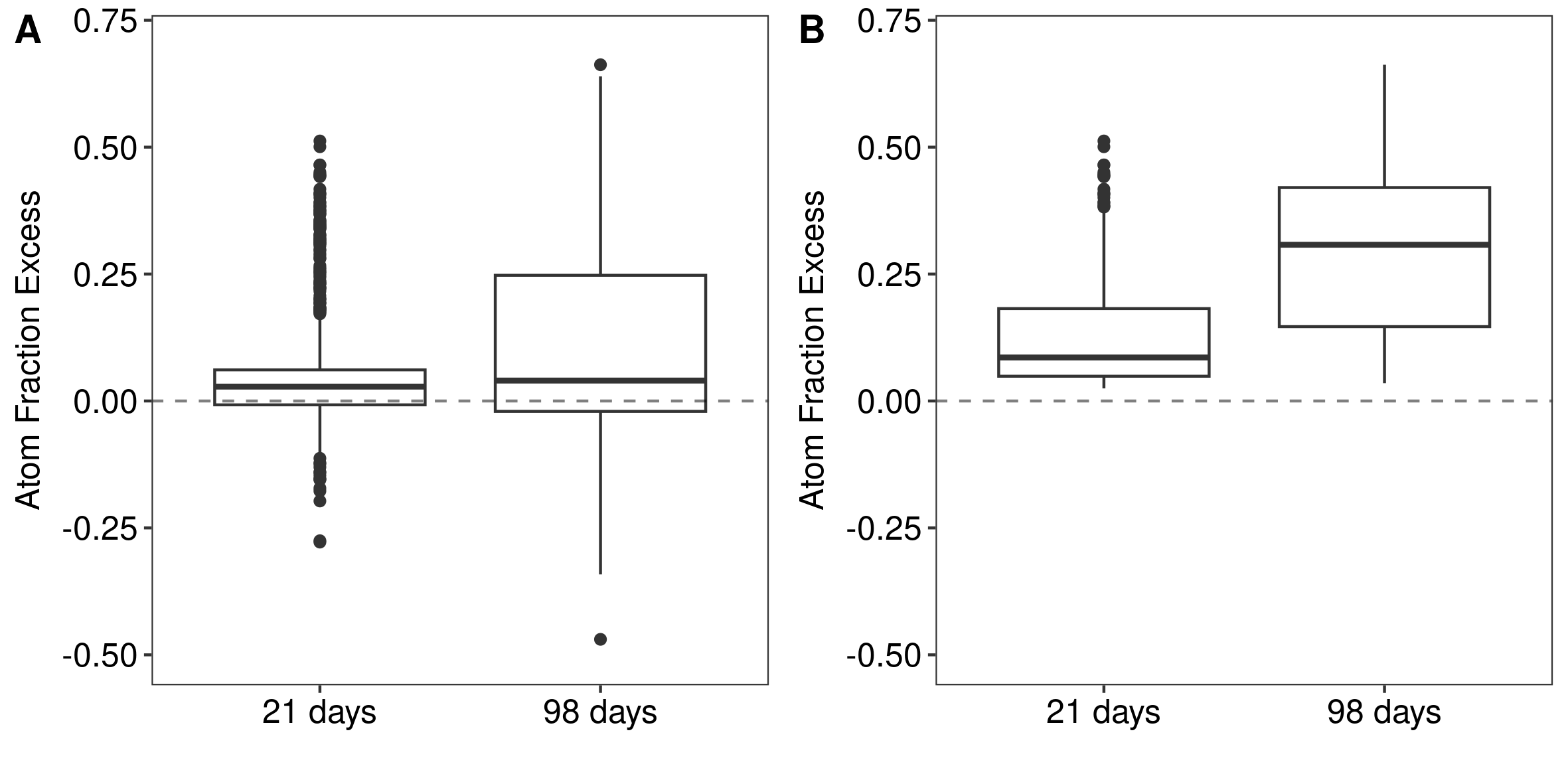
**

Fig. S5: Atom fraction excess (AFE) in DNA of ASVs. AFE shows the magnitude of incorporation of ^18^O into microbial DNA at 21 and 98 days under oxic conditions. A) AFE of all growing (^18^O-labeled) and non-growing (unlabeled) ASVs. B) AFE of only ^18^O-labeled ASVs with over 90% confidence.


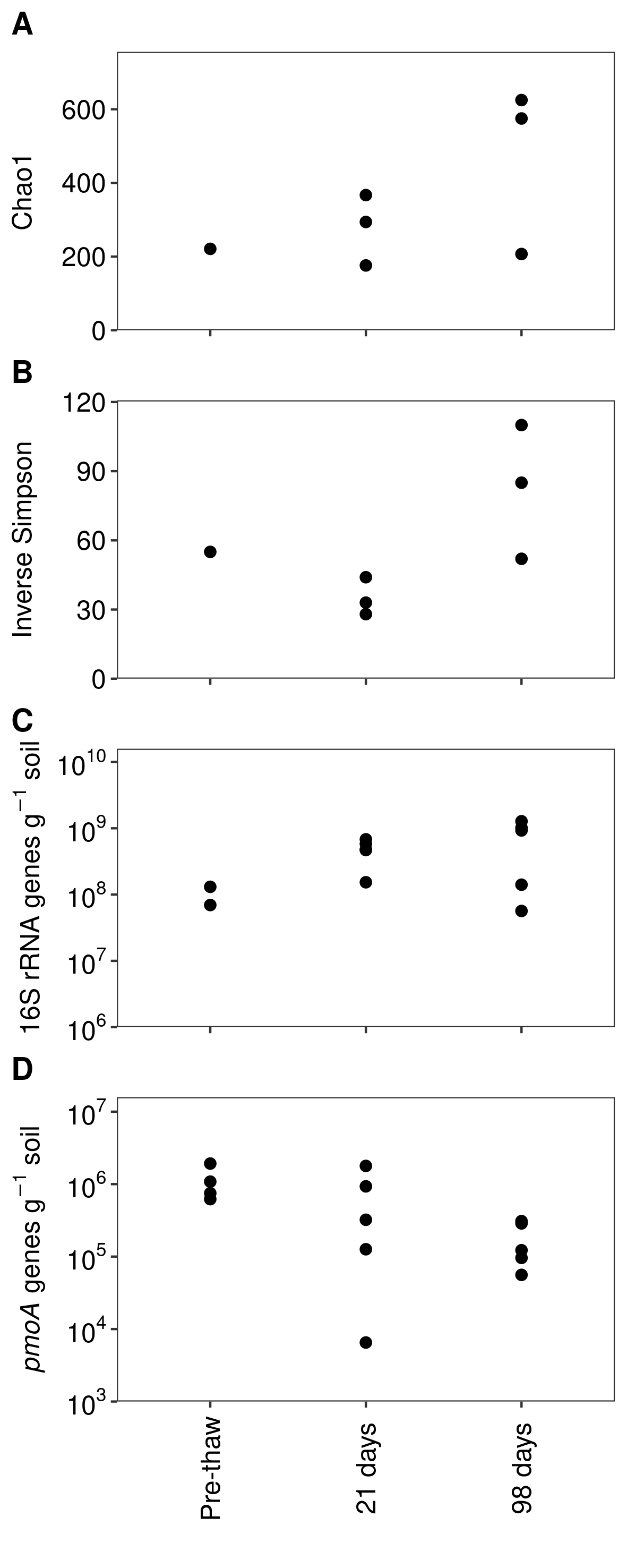


Fig. S6. Alpha diversity and gene abundance of unfractionated libraries before (pre-thaw) and after 21 and 98 days of thaw at 4 °C under oxic conditions. Alpha diversity index calculations were the mean of 10 iterations. A) Chao1 diversity index estimate of ASV richness. B) Inverse Simpson diversity index indicating richness and evenness. Evenness considers the abundance of each ASV additional to richness estimates. The Inverse Simpson Index increases in value with an increase in the number of ASVs and when ASVs are in similar abundance. Inverse Simpson Index is less influenced by rare ASVs. C) Abundance of 16S rRNA genes per g of wet soil before (pre-thaw) and after 21 and 98 days of thaw. D) Abundance of *pmoA* genes per g of wet soil before (pre-thaw) and after 21 and 98 days of thaw. Welch two-sample *t*-tests for changes in abundance of 16S rRNA genes, Chao1, and Inverse Simpson diversity were insignificant at p = 0.46, p = 0.29 and p = 0.10 respectively. Wilcoxon rank sum test for changes in abundance of *pmoA* genes were insignificant at p = 0.31)


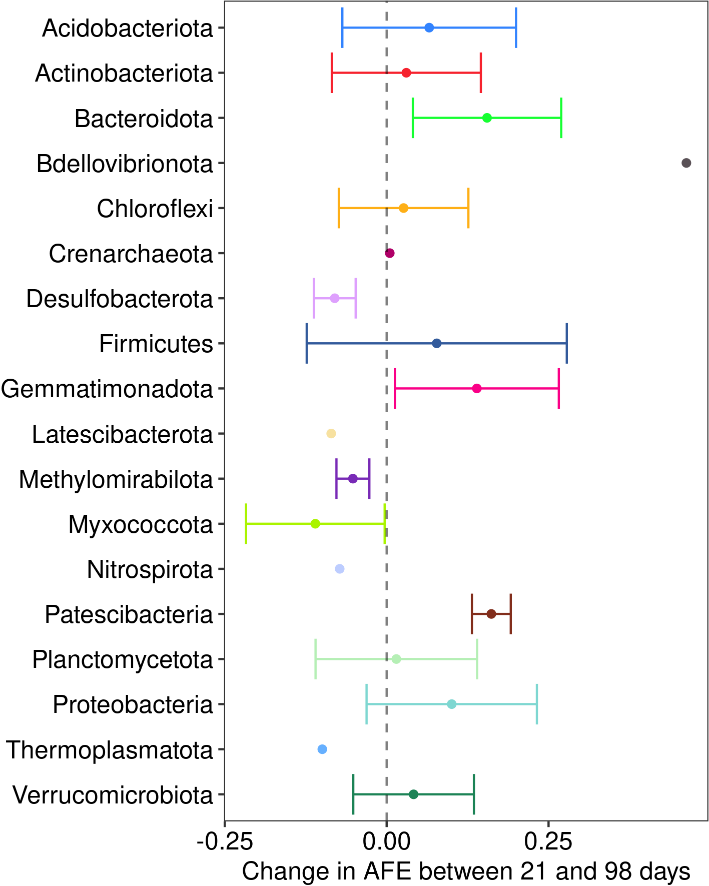


Fig. S7: ASV-specific increase in atom fraction excess (AFE) by phylum. Data shows the change in AFE between 21 and 98 days of thaw at 4°C for ASVs for which ^18^O-labeling occurred at 21 or 98 days under oxic conditions. The mean difference in AFE between 21 and 98 days for ASVs is grouped by phyla. Error bars represent one standard deviation from the mean.


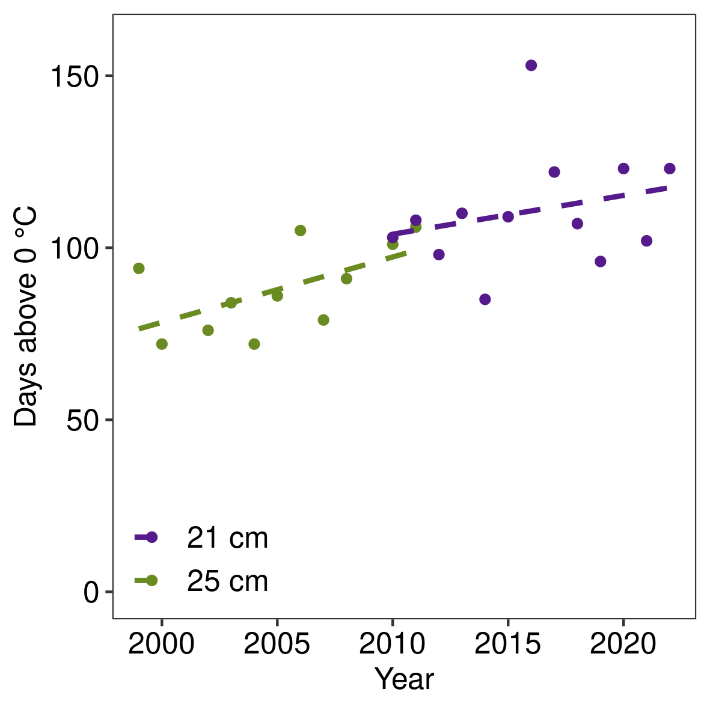


Fig. S8: Annual number of days above 0 °C. The number of days each year that noon temperature of soil at 21 cm (purple) or 25 cm (green) depth rises above 0 °C is shown. The data was collected from Boike et al. (2022). Dashed lines represent linear models for the 21 cm (adjusted R^2^ = 0.98, p = 1.9 × 10^-11^) and 25 cm (adjusted R^2^ = 0.98, p = 4.7 × 10^-10^)

Supplementary Tables

Table S1: List of physical and biological characteristics of active layer soil from 18 to 27 cm depth near the Bayelva Permafrost Monitoring Observatory near Ny-Ålesund, Svalbard (soil core). Concentration of trace elements in active layer soil from 20 to 25 cm depth near the Bayelva Permafrost Monitoring Observatory near Ny-Ålesund, Svalbard (soil pit). Elements were measured using ICP-MS. Concentration units are mg Kg^-1^.

| Soil core.  Location: 78.921078° N, 11.857037° E  Depth: 18–27 cm | Gravimetric soil moisture (%) | | 10.3 ± 0.14 |
| --- | --- | --- | --- |
|  | Organic matter content (%) | | 2.5 ± 0.12 |
|  | Cell count (cells g^-1^) | | 2.6 ± 1.8 × 10^6^ |
|  | Total DNA concentration (ng DNA g^-1^ soil) | | 45 ± 28 |
| Soil pit.  Location: 78.921367° N, 11.865867° E  Depth: 20–25 cm | Trace elements (mg Kg^-1^) | Mg | 4580 |
|  |  | Mn | 478 |
|  |  | Fe | 23638 |
|  |  | Co | 13 |
|  |  | Ni | 23 |
|  |  | Cu | 16 |
|  |  | Zn | 46 |
|  | pH | | 6.05 |

Table S2: Mean atom fraction excess (AFE) and relative 16S rRNA gene abundance of dominant soil microbial phyla under oxic conditions. The standard deviation of AFE values of ASVs affiliated to each phylum is shown. The standard error in 16S rRNA gene relative abundance between microcosm replicates is shown.

|  | Atom fraction Excess (AFE) | | Relative 16S rRNA gene abundance (%) | |
| --- | --- | --- | --- | --- |
| Phylum | **21 days** | **98 days** | **21 days** | **98 days** |
| Acidobacteriota | 0.0794 ± 0.0364 | 0.188 ± 0.116 | 13.28 ± 1.17 | 11.33 ± 0.13 |
| Actinobacteriota | 0.0951 ± 0.0792 | 0.223 ± 0.124 | 33.71 ± 1.83 | 24.07 ± 0.89 |
| Bacteroidota | 0.268 ± 0.115 | 0.370 ± 0.102 | 1.91 ± 0.56 | 10.95 ± 1.58 |
| Proteobacteria | 0.238 ± 0.134 | 0.386 ± 0.128 | 21.10 ± 4.29 | 23.25 ± 1.62 |

Table S3: The relative sequence abundance (%) of the 16S rRNA gene amplicon sequencing library associated to ASVs that were ^18^O-labeled*, unlabeled, negatively labeled, and not detected by the qSIP** method.

|  | Relative abundance (%) of ASVs | |
| --- | --- | --- |
|  | 21 days | 98 days |
| ^18^O-labeled, growing | 50.6 ± 7.4 | 53.8 ± 4.2 |
| Unlabeled, not growing | 27.9 ± 6.7 | 29.0 ± 3.3 |
| Negatively labeled | 1.0 ± 0.8 | 0.9 ± 0.8 |
| Not detected by qSIP | 21.8 ± 4.7 | 17.2 ± 1.0 |

* The relative sequence abundance belonging to ^18^O-labeled ASVs is not the amount of total ^18^O-labeled DNA since within a single ASV population there may be growing and non-growing cells.

**The “Not detected by qSIP” values are the relative sequence abundance for which ^18^O-labeling cannot be determined because these groups were not found in both the H_2_^18^O-amended and H_2_^16^O control fractionated replicate libraries. These “not detected by qSIP” values represent the non-reproducible portion of the replicate sequencing libraries.

References

Boike, J., Grünberg, I., Miesner, F., Bornemann, N. and Cable, W. L., 2022. Continuous measurements in soil and air at the permafrost long-term observatory at the Bayelva station near Ny-Ålesund (2018 et seq). [online]. Available from: https://doi.pangaea.de/10.1594/PANGAEA.948951 [Accessed 24 Jan 2024].

Correggia, M., Di Iorio, L., Bastianoni, A. B., Yücel, M., Cordone, A. and Giovannelli, D., 2023. Standard Operating Procedure for the analysis of trace elements in hydrothermal fluids by Inductively Coupled Plasma Mass Spectrometry (ICP-MS). *Open Research Europe*, 3, 90.

Coskun, Ö. K., Pichler, M., Vargas, S., Gilder, S. and Orsi, W. D., 2018. Linking Uncultivated Microbial Populations and Benthic carbon turnover by using quantitative stable isotope probing. *Applied and Environmental Microbiology*, 84 (18), 1–15.

Hay Mele, B., Monticelli, M., Leone, S., Bastoni, D., Barosa, B., Cascone, M., Migliaccio, F., Montemagno, F., Ricciardelli, A., Tonietti, L., Rotundi, A., Cordone, A. and Giovannelli, D., 2023. Oxidoreductases and metal cofactors in the functioning of the earth. *Essays in Biochemistry*, 67 (4), 653–670.

Khalili, B., Weihe, C., Kimball, S., Schmidt, K. T. and Martiny, J. B. H., 2019. Optimization of a Method To Quantify Soil Bacterial Abundance by Flow Cytometry. *mSphere*, 4 (5), e00435-19.
