# Supplementary material for: Seasonal thawing of high Arctic soils triggers selective microbial growth and predation
Source: mSystems. 2026 May 7;11(6):e00738-25. doi: 10.1128/msystems.00738-25 (PMC13288927; doi:10.1128/msystems.00738-25)
Supplement: Reviewer comments [file reviewer-comments.pdf]

AMERICAN SOCIETY FOR MICROBIOLOGY | mSystems®

Seasonal thawing of high Arctic soils triggers selective microbial growth and predation

Margaret Cramm, Ömer Kürsat Coskun, Francesco Montemagno, Matteo Selci, Daniel Read, Tim Goodall, Brianna Green, Sayali Mulay, Katie Sipes, Andrey Abramov, Catherine Heppell, Julia Boike, Donato Giovannelli, Tatiana Vishnivetskaya, Robert Hettich, Andrew Steen, Karen Lloyd, William Orsi, Anne Jungblut, and James Bradley

Corresponding Author(s): Margaret Cramm, Queen Mary University of London

Review Timeline:

Submission Date:	May 22, 2025
Editorial Decision:	July 29, 2025
Revision Received:	September 26, 2025
Editorial Decision:	December 18, 2025
Revision Received:	February 9, 2026
Editorial Decision:	March 4, 2026
Revision Received:	March 18, 2026
Accepted:	March 19, 2026

Editor: Emily Graham

Reviewer(s): Disclosure of reviewer identity is with reference to reviewer comments included in decision letter(s). The following individuals involved in review of your submission have agreed to reveal their identity: Bram W. G. Stone (Reviewer #1); Zahra Fatima Islam (Reviewer #2)

Transaction Report:

(Note: With the exception of the correction of typographical or spelling errors that could be a source of ambiguity, letters and reports are not edited. The original formatting of letters and referee reports may not be reflected in this compilation.)

DOI: https://doi.org/10.1128/msystems.00738-25

Re: mSystems00738-25 (Seasonal thawing of high Arctic soils triggers selective microbial growth and predation)

Dear Dr. Margaret Anne Cramm:

Thank you for the privilege of reviewing your work. Below you will find my comments, instructions from the mSystems editorial office, and the reviewer comments.

Please pay particular attention to the methodological issues raised, including clarification on replication and sampling.

Please return the manuscript within 60 days; if you cannot complete the modification within this time period, please contact me. If you do not wish to modify the manuscript and prefer to submit it to another journal, notify me immediately so that the manuscript may be formally withdrawn from consideration by mSystems.

Revision Guidelines
To submit your modified manuscript, log into the submission site at https://msystems.msubmit.net/cgi-bin/main.plex. Go to Author Tasks and click the appropriate manuscript title to begin. The information you entered when you first submitted the paper will be displayed; update this as necessary. Note the following requirements:

• Upload point-by-point responses to the issues raised by the reviewers in a file named "Response to Reviewers," NOT in your cover letter.
• Upload a compare copy of the manuscript (without figures) as a "Marked-Up Manuscript" file.
• Upload a clean .DOC/.DOCX version of the revised manuscript and remove the previous version.
• Each figure must be uploaded as a separate, editable, high-resolution file (TIFF or EPS preferred), and any multipanel figures must be assembled into one file.
• Any supplemental material intended for posting by ASM should be uploaded with their legends separate from the main manuscript. You can combine all supplemental material into one file (preferred) or split it into a maximum of 10 files with all associated legends included.

For complete guidelines on revision requirements, see our Submission and Review Process webpage. Submission of a paper that does not conform to guidelines may delay acceptance of your manuscript.

Data availability: ASM policy requires that data be available to the public upon online posting of the article, so please verify all links to sequence records, if present, and make sure that each number retrieves the full record of the data. If a new accession number is not linked or a link is broken, provide mSystems production staff with the correct URL for the record. If the accession numbers for new data are not publicly accessible before the expected online posting of the article, publication may be delayed; please contact production staff (mSystems@asmusa.org) immediately with the expected release date.

Publication Fees: For information on publication fees and which article types are subject to charges, visit our website. If your manuscript is accepted for publication and any fees apply, you will be contacted separately about payment during the production process; please follow the instructions in that e-mail. Arrangements for payment must be made before your article is published.

ASM Membership: Corresponding authors may join or renew ASM membership to obtain discounts on publication fees. Need to upgrade your membership level? Please contact Customer Service at Service@asmusa.org.

The ASM Journals program strives for constant improvement in our submission and publication process. Please tell us how we can improve your experience by taking this quick Author Survey.

Thank you for submitting your paper to mSystems.

Sincerely,
Emily Graham
Editor
mSystems

Reviewer #1 (Comments for the Author):

Overall comments:
This study examines the growth of microorganisms from high Arctic soils subjected to laboratory thawing using 18O-water qSIP. The depth of the soils was the active subsurface which was 18-27 below the surface. The main findings were of a greater diversity of growing organisms 98 days following thaw (vs 24 days) and an interesting finding of enrichment among organisms containing the pmoA gene necessary for methane oxidation. The authors discuss the biology and possible biological roles of

these organisms based on major taxonomic groupings.

My main concerns are with the size and replication of the study which appears to be minimal or non-existent. The authors present 3 labeled samples in figures but it seems like they created a single labeled microcosm for each timepoint, and so this would be more akin to a technical replicate. However this is hard to verify from the text as written (are these replicates from the soil from the core or replicates from the same DNA at the spinning stage?). The authors need to clarify where and how these replicates arise more consistently across the methods so that readers don't have to jump back and forth through the methods to piece together their scheme. It's also unclear why they analyzed the weighted buoyant densities from their non-labeled samples separately from each timepoint. In theory, the non-labeled buoyant density of the organisms should be unchanged, thus combining them produces a greater index of non-labeled densities for all organisms and potentially allowing a greater number of enrichment values to be resolved (and decreasing the uncertainty of the bootstrap enrichment estimate of the day 98 timepoint. Did the authors separate the non-labeled samples to control for potential biases of each spin? I would recommend running through the analysis again with a combined non-labeled density index and seeing if this reduces uncertainty.

Specific comments:

L151-513: How long were soils stored at 20C prior to 4C incubation?

L157-158: Can some of the relevant features of the bulk soil be reported in the results?

L162-164: What does immediately mean, does this mean samples were aliquoted in to 2 g vials immediately after thawing? How was this determined? What was the duration of time that the collected soil was at 20C? How much total soil was collected? Was it mixed prior to distributing to vials? Did all vials receive soil fro a single core?

L164: What is the total atom % 18O of the 18O-water? Please reconsider the terminology for 16O-water. It is not entirely free of 18O atoms and should be clarified as water at natural abundance 18O -- and then the authors may designate an H2^16^O term as a shorthand if they wish. I see these details are indicated on line 170 and should be moved up.

L172-L173: I don't think this statement is appropriate -- there are several aspects of this experiment that do not align with the Hungate article. It would be more appropriate to cite the Trejos-Espeleta article for previous use of the slurry approach only. The difference in experiment duration (1 and 7 days from Trejos-Espeleta vs 21 and 98 days here) is drastic and the use of 13C amino acids vs 18O water is another significant difference.

L173-174: I am concerned about the creation of slurries in the soils using 2:1 w/v (water/soil) as it is not clear whether this type of saturation is natural for the soils. The authors should include more details of the native soil conditions here so readers can evaluate how much this may or may not depart from natural conditions.

L176-177: What is the purpose of the anoxic chambers? What measurements were made from these?

L219-221: Which fractions were selected for density measurement? This is unusual with respect to previous studies and the authors should show the measured and modeled densities as supplemental data.

L269: Which mesocosms were anoxic? How was this determined? Does this mean some vials failed to be kept oxic or that the in-parallel anoxic chambers were not considered for qSIP?

L270-288: How many ASVs were detected total? How many excluding global singletons and doubletons?

L281-282: Why only ten iterations? 100-1000 iterations would be better for rarefied alpha diversity.

L284-288: Please clarify -- the data are rarefied for alpha diversity metrics but not for beta diversity? Were rarefied data used only for alpha diversity and no other analysis?

L294-296: What were the numbers of technical replicates created?

L292-305: How many ASVs had a measurable enrichment value? The authors claim that negative enrichment values may arise from variability in rare ASVs -- was this verified? The authors should perform a test e.g., whether the median abundance from "negative enrichment" organisms lower than the median abundance of "positive enrichment" organisms (or at least visualize the overlap).

L301-303: Why were the 16O-water control replicates kept separate? The buoyant densities of DNA from all control samples should be the same and they could be grouped together. If this tightens the enrichment confidence interval and results in a greater number of enriched taxa, the results should be updated. If the authors elect to keep these separate, they should

describe their analytical rationale for doing so in the methods.

L342-343: While technically accurate this is misleading as the earliest timepoint with which to make the observation for growth is 21 days, even if respiration was measured sooner. Please adjust.

L353-255: This interpretation is not supported by the results presented up to this point as the composition of "growers" has not been shown. If this interpretation fits results that are shown further down, it should be moved to that area. If no other data are shown to support this statement, it should be removed. From the very next sentences, it seems they are of similar composition and diversity (i.e., not distinctly "late" or "early' communities).

L425-426: The statement that "early" and "late" growing communities are distinct contradicts the previous findings (L356-363).

L425-443: A longer incubation with 13C sources will also allow for a greater diversity of enriched taxa simply through cross-feeding. How can interpretations based on life strategy be separated from this artifact?

L439-440: Please cite literature, especially for the idea that slow growth is advantageous for changing environments.

L445: Either the predator section should called out here, or the labels of "predation, and death" should be reduced to simply "turnover". Given that this paragraph is simply describing the dynamics of different lineages at different timepoints, I think the latter option is more suitable.
L452-455: This statement could be strengthened as above, especially considering the high enrichment of predatory lineages.
L471-498: The Hungate paper merits citing here: Hungate et al. 2021. https://doi.org/10.1128/mbio.00466-21

L489: Please briefly define epibiosis and specify how it relates to the lineages discussed above. This link isn't clear from the information presented.

L520-523: This is hard to understand, please clarify. Figure 5 shows a relativized abundance. Is this statement referring to the bi-modality of density curves in 5B? The interpretation that "roughly half" the organisms capable of methane oxidation grew must be clarified -- either some populations group and some didn't or every population contains growing and non growing cells. Which did the authors mean? Regardless, neither can be determined with the data as currently presented at this point in the paper. The authors should use another wording besides "organisms" to help convey this limitation.

L553-556: This strikes me as an over interpretation given the lack of biological replicates in this experiment.

L569-570: This claim is also an over interpretation given the data included.

Figure 2: What do the solid and dashed lines represent? How does this relate to the experimental design -- are these the technical replicates?

Figure 5: As in figure 2, what do the solid and dashed lines represent?

Supplemental Table 1: As indicated earlier, please consider importing these basic descriptive analyses to the description of the site in the methods so that readers can understand the site and context.

Supplemental Table 1: As indicated earlier, please consider importing these basic descriptive analyses to the description of the site in the methods so that readers can understand the site and context.

Reviewer #2 (Comments for the Author):

Overall comments:
Overall, the manuscript by Cramm et al., provided a very interesting insight into the temporal patterns of microbial growth following thawing of Arctic permafrost through the use of DNA SIP. The manuscript provided good evidence to highlight taxon-specific growth dynamics of thawed soil microcosms over a prolonged time period, with clear evidence indicating that microbial communities shift over time, and that predatory bacterial taxons appear to benefit from this seasonal thawing process, which is likely to accelerate with a changing global climate. Overall, the manuscript was well-written and well-formatted, but there are a couple of methodological clarifications that should be addressed prior to the manuscript being accepted for publication.

Major comments:
L129: What is the specific relevance of the 21 and 98 day timepoints? Do these timings correspond to specific atmospheric/climactic changes in the natural environment? As the soils were not used straight away after collection, it is unclear

how the timepoints chosen align with the environmental temperature data shown in Figure 1D.
L152: What was the rationale behind only performing the trace element analysis on soils collected on a different pit at a completely different time (March 2021 for microcosms, June 2022 for trace element analysis)? Whilst it may be possible that there are very limited changed in soil physicochemical properties over the course of 1.25 years, this cannot be definitively ruled out.
L174-176: How many times over the incubation period was the headspace in the microcosms replaced? If the headspace wasn't replaced, this would likely lead to the vials becoming anoxic, particularly by the 98 day timepoint, which would likely culminate in shifts in microbial community composition. As the thawing soils would likely be exposed to a 'normal' atmosphere with regular gas flux, only adding in oxygen at the start of the incubation period, and using air without CH_4 or CO_2, may not accurately reflect the natural environment of the soils, and thus would alter the microbial community composition over the incubation period.
L444-467: Did you take a look into lower taxonomic ranks, or just phylum level data? This would be particularly helpful for ascribing functions to the different microbial communities, as generalising bacterial functions on a phylum level is not particularly informative.
L511-546: Given the absence of methane in the headspace of the microcosms, and the lack of methanogenic species identified within the microbial communities, it seems likely that there would only be a negligible increase in methanotroph populations, unless they were mixotrophic? As mentioned before, a complete lack of methane in the microcosm headspace would also not be particularly representative of the natural environment, where methane would be present in trace amounts (similar to that of H_2 and CO, as mentioned in L568).
Supplementary Figure 2: There is a huge spike in CH_4 concentration in one of the day 21 microcosm replicates. Is there any explanation for this? And if so, how would this affect the community composition of that replicate compared to the others in the set? Additionally, is there seems to be a clear decrease in CO_2 concentration for 3 out of 5 replicates for the 98 day microcosm, suggesting that these vials are no longer actively growing.

Minor comments:
L123-124: Missing word "to" - ".... glacial forefield soils that were sensitive to successional stage and local vegetation."
L416-420: In these lines, three bacterial phyla are mentioned, but only two numbers are quoted, so it is unclear which phyla correspond to which number as there is one missing.
L546: Missing reference to manuscript Figure where data has arisen from.
Supplementary Table 1: The layout of this table is confusing, as it currently suggests that trace elements were only measured in the 20-24cm depth profile, as opposed to the other metrics which were only measured in the 18-27cm depth profile? Additionally, was only one sample used to calculate the cell count and total DNA concentration, as there are no SDs reported?

Reviewer comments

Manuscript number: mSystems00738-25

Article title: Seasonal thawing of high Arctic soils triggers selective microbial growth and predation

Article type: Original research

Overall comments:

Overall, the manuscript by Cramm et al., provided a very interesting insight into the temporal patterns of microbial growth following thawing of Arctic permafrost through the use of DNA SIP. The manuscript provided good evidence to highlight taxon-specific growth dynamics of thawed soil microcosms over a prolonged time period, with clear evidence indicating that microbial communities shift over time, and that predatory bacterial taxons appear to benefit from this seasonal thawing process, which is likely to accelerate with a changing global climate. Overall, the manuscript was well-written and well-formatted, but there are a couple of clarifications that should be addressed prior to the manuscript being accepted for publication.

Major comments:

L129: What is the specific relevance of the 21 and 98 day timepoints? Do these timings correspond to specific atmospheric/climactic changes in the natural environment? As the soils were not used straight away after collection, it is unclear how the timepoints chosen align with the environmental temperature data shown in Figure 1D.

L152: What was the rationale behind only performing the trace element analysis on soils collected on a different pit at a completely different time (March 2021 for microcosms, June 2022 for trace element analysis)? Whilst it may be possible that there are very limited changed in soil physicochemical properties over the course of 1.25 years, this cannot be definitively ruled out.

L174-176: How many times over the incubation period was the headspace in the microcosms replaced? If the headspace wasn't replaced, this would likely lead to the vials becoming anoxic, particularly by the 98 day timepoint, which would likely culminate in shifts in microbial community composition. As the thawing soils would likely be exposed to a 'normal' atmosphere with regular gas flux, only adding in oxygen at the start of the incubation period, and using air without CH4 or CO2, may not accurately reflect the natural environment of the soils, and thus would alter the microbial community composition over the incubation period.

L444-467: Did you take a look into lower taxonomic ranks, or just phylum level data? This would be particularly helpful for ascribing functions to the different microbial communities, as generalising bacterial functions on a phylum level is not particularly informative.

L511-546: Given the absence of methane in the headspace of the microcosms, and the lack of methanogenic species identified within the microbial communities, it seems likely that there would only be a negligible increase in methanotroph populations, unless they were mixotrophic? As mentioned before, a complete lack of methane in the microcosm headspace would also not be particularly representative of the natural environment, where methane would be present in trace amounts (similar to that of H2 and CO, as mentioned in L568).

Supplementary Figure 2: There is a huge spike in CH4 concentration in one of the day 21 microcosm replicates. Is there any explanation for this? And if so, how would this affect the

community composition of that replicate compared to the others in the set? Additionally, is there seems to be a clear decrease in CO_2 concentration for 3 out of 5 replicates for the 98 day microcosm, suggesting that these vials are no longer actively growing.

Minor comments:

L123-124: Missing word “to” – “…. glacial forefield soils that were sensitive **to** successional stage and local vegetation.”

L416-420: In these lines, three bacterial phyla are mentioned, but only two numbers are quoted, so it is unclear which phyla correspond to which number as there is one missing.

L546: Missing reference to manuscript Figure where data has arisen from.

Supplementary Table 1: The layout of this table is confusing, as it currently suggests that trace elements were only measured in the 20-24cm depth profile, as opposed to the other metrics which were only measured in the 18-27cm depth profile? Additionally, was only one sample used to calculate the cell count and total DNA concentration, as there are no SDs reported?

Response to editor and reviewers' comments on 'Seasonal thawing of high Arctic soils triggers selective microbial growth and predation (mSystems00738-25)

<u>EDITOR</u>

Thank you for the privilege of reviewing your work. Below you will find my comments, instructions from the mSystems editorial office, and the reviewer comments. Please pay particular attention to the methodological issues raised, including clarification on replication and sampling.

We would like to thank the editor and reviewers for their very helpful and constructive comments and suggestions, which have helped improve the manuscript considerably. We have addressed all the suggestions that were raised, including the methodological issues raised by the reviewers, and clarification on replication and sampling. Please find below our responses to specific points.

Please note that the line numbers are correct according to the marked up version of the document when track changes are visible to the reader.

<u>REVIEWER #1</u>

Overall comments:
This study examines the growth of microorganisms from high Arctic soils subjected to laboratory thawing using 18O-water qSIP. The depth of the soils was the active subsurface which was 18-27 below the surface. The main findings were of a greater diversity of growing organisms 98 days following thaw (vs 24 days) and an interesting finding of enrichment among organisms containing the pmoA gene necessary for methane oxidation. The authors discuss the biology and possible biological roles of these organisms based on major taxonomic groupings.

My main concerns are with the size and replication of the study which appears to be minimal or non-existent. The authors present 3 labeled samples in figures but it seems like they created a single labeled microcosm for each timepoint, and so this would be more akin to a technical replicate. However this is hard to verify from the text as written (are these replicates from the soil from the core or replicates from the same DNA at the spinning stage?). The authors need to clarify where and how these replicates arise more consistently across the methods so that readers don't have to jump back and forth through the methods to piece together their scheme. It's also unclear why they analyzed the weighted buoyant densities from their non-labeled samples separately from each timepoint. In theory, the non-labeled buoyant density of the organisms should be unchanged, thus combining them produces a greater index of non-labeled densities for all organisms and potentially allowing a greater number of enrichment values to be resolved (and decreasing the uncertainty of the bootstrap enrichment estimate of the day 98 timepoint. Did the authors separate the non-labeled samples to control for potential biases of each spin? I would recommend running through the analysis again with a combined non-labeled density index and seeing if this reduces uncertainty.

Thank you for your detailed review of our manuscript. We have incorporated your recommendations in the revised manuscript as detailed below. In particular, the description of the challenges you experienced regarding understanding the study replication have been very useful in improving the clarify of our manuscript. These have now been addressed, as per the comments below and herein. We have made changes throughout the manuscript, particularly in the Methods section, to make clearer the biological and technical replication in this study. In short,

- All soils were from the same soil core.
- Analyses of the whole community composition in incubations were done with 3 distinct microcosm incubations. Analyses of ^{18}O incorporation into DNA was done through the comparison of a single labelled microcosm and a single unlabelled microcosm.
- Differences in biological replication are an inherent variable within the calculation of ^{18}O incorporation, which relies on pairwise ASV-ASV comparisons between the two biological replicates and was calculated at ~20% for this study. This is the same method that was used by Trejos-Espeleta et al. (2024)
- Technical triplicates of DNA fractionation were done to determine variability in DNA fractionation technique, including biases in DNA centrifugation as you have identified above in your review. This is the same method that was used by Trejos-Espeleta et al. (2024).

We have modified the revised manuscript to make these details clear (see detailed points below for specific modifications).

Specific comments:

L151-513: How long were soils stored at 20C prior to 4C incubation?
The soils were collected 25 March 2021 and stored at -20 °C for 3 months. We have added this information to the revised manuscript (line 168).

L157-158: Can some of the relevant features of the bulk soil be reported in the results?

We have moved the description of bulk soil characteristics from the Supplementary Information to the Results and Discussion sections (lines 342-350).

L162-164: What does immediately mean, does this mean samples were aliquoted in to 2 g vials immediately after thawing? How was this determined? What was the duration of time that the collected soil was at 20C? How much total soil was collected? Was it mixed prior to distributing to vials? Did all vials receive soil fro a single core?

All vials received soil from a single core since this was the only core retrieved for microbiological sampling at this site. We collected a total of 343 g of soil, but only ~100 g was thawed for creating microcosms. Two grams of soil were aliquoted into vials (using a scale) as soon as soil-bound ice became liquid, forming an ice-free water-soil slurry. This

was carried out under real-time visual monitoring and took approximately 1 hour from the moment soils were withdrawn from -20°C into a laboratory at room temperature. The slurry was homogenized and then immediately distributed into vials which were placed on ice packs during microcosm set-up (to maintain a cold temperature of the sample). The slurry (whilst on ice) was subjected to the +20°C of the lab environment for up to 20 min prior to incubation at 4°C. We have highlighted these details in lines 163 and 185 for enhanced clarity.

L164: What is the total atom % 18O of the 18O-water? Please reconsider the terminology for 16O-water. It is not entirely free of 18O atoms and should be clarified as water at natural abundance 18O -- and then the authors may designate an H2^16^O term as a shorthand if they wish. I see these details are indicated on line 170 and should be moved up.

We have rearranged this paragraph to be clearer regarding the water compositions used in microcosm creation (lines 185-186).

L172-L173: I don't think this statement is appropriate -- there are several aspects of this experiment that do not align with the Hungate article. It would be more appropriate to cite the Trejos-Espeleta article for previous use of the slurry approach only. The difference in experiment duration (1 and 7 days from Trejos-Espeleta vs 21 and 98 days here) is drastic and the use of 13C amino acids vs 18O water is another significant difference.

Thanks for this comment. We have amended this statement in the text to be precise about the elements of experimental design mirroring Trejos-Espeleta et al. (2024) (i.e. replication with regard to DNA-SIP methods) and the aspects of our experimental design that are different (including, as the reviewer notes, the use of $H_2{}^{18}O$ rather than ^{13}C amino acids as a label) (lines 196-198).

L173-174: I am concerned about the creation of slurries in the soils using 2:1 w/v (water/soil) as it is not clear whether this type of saturation is natural for the soils. The authors should include more details of the native soil conditions here so readers can evaluate how much this may or may not depart from natural conditions.

Thank you for highlighting this caveat. We aimed for a 2:1 w/v water soil ratio for slurry creation in order to obtain a water composition of >60% ^{18}O, which is desirable for the qSIP method. We address this caveat (i.e. the excess water added to create microcosm slurries) in lines 696-697 under the heading “Changing hydrology”. We have added the following sentence to clarify soil moisture increases in the microcosms compared to that of the in situ soil: “This led to a 5 times increase in soil moisture in microcosms compared to the collected soil, which is slightly higher than the 3 times soil moisture increase that happens in situ seasonal soil thaw (Grünberg et al. 2024)”. (lines 199-202)

L176-177: What is the purpose of the anoxic chambers? What measurements were made from these?

The anoxic microcosms were used to explore possible methane production under an anoxic regime and compare it to the possible methane production in oxic microcosms. They were also used to compare overall community composition differences between oxic and anoxic regimes – which we briefly mention in lines 625-626. The detection of growth using qSIP methods was not carried out on anoxic microcosms – rather, we only report gas composition measurements from the anoxic microcosms and reference whole community composition.

L219-221: Which fractions were selected for density measurement? This is unusual with respect to previous studies and the authors should show the measured and modeled densities as supplemental data.

The density of fractions 1-4, 8, and 17-20 were measured using the refractometer, and the density of the remaining fractions was inferred based on a linear model. Our approach follows the methods used in Coskun et al. (2018) and Trejos-Espeleta et al. (2024). Measuring the density using the refractometer is destructive, and thus our approach was used to minimize the loss of density gradient solutions. We have added a supplementary figure showing the measured and inferred densities (Supplementary Figure 1).

L269: Which mesocosms were anoxic? How was this determined? Does this mean some vials failed to be kept oxic or that the in-parallel anoxic chambers were not considered for qSIP?

We created oxic (headspace flushed with zero grade air) and anoxic (headspace flushed with zero grade nitrogen gas) microcosms in order to test different conditions. The headspace gas of the incubations were not replaced during the incubations. We have added a sentence indicating this in line 215-216 and removed the sentence in line 298 and in line 330, for improved clarity in the revised manuscript.

L270-288: How many ASVs were detected total? How many excluding global singletons and doubletons?

There were 2,666 ASVs in the dataset with 40 ASVs being singletons and 97 doubletons. This information has been added in lines 308-310.

L281-282: Why only ten iterations? 100-1000 iterations would be better for rarefied alpha diversity.

Thank you for this suggestion. We re-did the alpha diversity measurements under 10, 100, and 1,000 iterations. The variance in the calculated diversity metrics under the different iterations was < 1 confirming that 10 iterations is suitable to accurately and precisely measure alpha diversity in these samples.

L284-288: Please clarify -- the data are rarefied for alpha diversity metrics but not for beta diversity? Were rarefied data used only for alpha diversity and no other analysis?

The reviewer’s interpretation is correct. Data were rarefied for the alpha diversity metrics only. To confirm that unrarefied data was suitable for beta diversity analyses, we did the ANOSIM test on both rarefied and non-rarefied distance matrices. The statistics of both are similar with the difference in the compared groups being insignificant ($\alpha = 0.05$) for both rarefied and non-rarefied data.

L294-296: What were the numbers of technical replicates created?

There were 3 technical replicates for the control (16-O) and 18O amended replicates. We have added this in line 242 for clarity.

L292-305: How many ASVs had a measurable enrichment value? The authors claim that negative enrichment values may arise from variability in rare ASVs -- was this verified? The authors should perform a test e.g., whether the median abundance from "negative enrichment" organisms lower than the median abundance of "positive enrichment" organisms (or at least visualize the overlap).

Thank you for raising this. We appreciate the suggestion of including values for instances of “negative labelling”. 43 of the 1044 ASVs in the 21-day incubations were negatively labelled and the relative abundance of these negatively labelled ASVs was 0.24 to 1.83%. 109 of the 1365 ASVs in the 98-day incubations were negatively labelled, corresponding to 0.35 to 1.78% of the relative sequence abundance in these incubations. We have modified Supplementary Table 3 to include this information.

There are far fewer negatively labelled ASVs than positively labelled ASVs. 326 of the 1044 ASVs in the 21-day incubations were positively labelled, corresponding to 42.5 to 57.0% of the relative sequence abundance in these incubations. 731 of the 1365 ASVs in the 98-day incubations were positively labelled corresponding to 50.1 to 58.4% of the relative sequence abundance in these incubations.

Given the large difference in the positively and negatively labelled ASVs’ abundance we think it is reasonable to attribute negatively labelled ASVs as a consequence of error related to low abundance ASVs - as is suggested by Sieradzki et al. (2020)(line 330).

L301-303: Why were the 16O-water control replicates kept separate? The buoyant densities of DNA from all control samples should be the same and they could be grouped together. If this tightens the enrichment confidence interval and results in a greater number of enriched taxa, the results should be updated. If the authors elect to keep these separate, they should describe their analytical rationale for doing so in the methods.

The technical replicates of the control capture the variability in the fractionating procedure. As such, we have opted to include these in the AFE calculations as per Youngblut et al (2018). We have added a sentence in lines 331-332 to make this clear.

L342-343: While technically accurate this is misleading as the earliest timepoint with which to make the observation for growth is 21 days, even if respiration was measured sooner. Please adjust.

This has been adjusted (line 391).

L353-255: This interpretation is not supported by the results presented up to this point as the composition of "growers" has not been shown. If this interpretation fits results that are shown further down, it should be moved to that area. If no other data are shown to support this statement, it should be removed. From the very next sentences, it seems they are of similar composition and diversity (i.e., not distinctly "late" or "early' communities).

Thank you for highlighting this. This sentence has been removed.

L425-426: The statement that "early" and "late" growing communities are distinct contradicts the previous findings (L356-363).

We have amended lines 463, 465 and 476-477 to clarify that the early and late *growing* communities (i.e. 18O-labelled) were distinct. This is alongside the observation that the entire communities (i.e. growing + non-growing) were compositionally similar. This highlights the value of using the qSIP method for identifying differences in communities that are compositionally similar.

L425-443: A longer incubation with 13C sources will also allow for a greater diversity of enriched taxa simply through cross-feeding. How can interpretations based on life strategy be separated from this artifact?

^{18}O incorporation into DNA happens both directly from water in the environment and from carbon and energy sources (Richards and Boyer 1966), and so it can't be determined exactly where the ^{18}O in labelled DNA came from when measuring DNA density increases. In all cases it simply indicates growth under the incubation conditions. Throughout this manuscript we have considered increases in DNA density to be a metric for growth without attributing this growth to carbon or energy resources, from the label or from cross-feeding. Thus, either way, our conclusions remain robust.

L439-440: Please cite literature, especially for the idea that slow growth is advantageous for changing environments.

We have added citations in line 492 slow growth to low energy environments (Gudelj et al. 2010; Jørgensen and Marshall 2016; Bird et al. 2019). We have also added a citation showing that populations growing in fluctuating nutrition environments grow slower than those where nutrition is stable (Nguyen et al. 2021).

L445: Either the predator section should called out here, or the labels of "predation, and death" should be reduced to simply "turnover". Given that this paragraph is simply describing the dynamics of different lineages at different timepoints, I think the latter option is more suitable.

This recommendation has been incorporated in lines 498 and 507-510.

L452-455: This statement could be strengthened as above, especially considering the high enrichment of predatory lineages.

Thank you for this suggestion. We have modified the statement to improve clarity.

L471-498: The Hungate paper merits citing here: Hungate et al.
2021. https://doi.org/10.1128/mbio.00466-21

We have added this citation to this section (line 545).

L489: Please briefly define epibiosis and specify how it relates to the lineages discussed above. This link isn't clear from the information presented.

We have added the definition for epibiosis (line 533).

L520-523: This is hard to understand, please clarify. Figure 5 shows a relativized abundance. Is this statement referring to the bi-modality of density curves in 5B? The interpretation that "roughly half" the organisms capable of methane oxidation grew must be clarified -- either some populations group and some didn't or every population contains growing and non growing cells. Which did the authors mean? Regardless, neither can be determined with the data as currently presented at this point in the paper. The authors should use another wording besides "organisms" to help convey this limitation.

We have edited this sentence to add clarity (lines 568-569). This statement is referring to the bi-modality of the curves corresponding to the ^{18}O-amended microcosm DNA (yellow curves, figure 5) which shows up to half of the DNA abundance peaking within the same density range as the ^{16}O-control microcosm DNA (purple curves, Figure 5, i.e. ~1.690 g mL^{-1}). Abundances are normalized to the maximum value within the same replicate allowing for proportional comparisons of peaks within each replicate.

L553-556: This strikes me as an over interpretation given the lack of biological replicates in this experiment.

Thank you for this comment. We agree and have edited this sentence to a more cautious interpretation that specifies that we did not detect any growth under the experimental conditions (line 623).

L569-570: This claim is also an over interpretation given the data included.

Thank you for this comment. We agree and have edited this statement to suggest that dormancy or slow growth contributes to total diversity under the experimental conditions explored here (line 631).

Figure 2: What do the solid and dashed lines represent? How does this relate to the

experimental design -- are these the technical replicates?

The solid and dashed lines correspond to technical replicates. We have added this information to the Figure caption (line 387).

Figure 5: As in figure 2, what do the solid and dashed lines represent?

The solid and dashed lines correspond to technical replicates. We have added this information to the Figure caption (line 577).

Supplemental Table 1: As indicated earlier, please consider importing these basic descriptive analyses to the description of the site in the methods so that readers can understand the site and context.

Descriptions of the site have been moved to the results section (lines 342-350), as per the reviewer's earlier comment, to make the site context clearer in the main text.

REVIEWER #2

Overall comments:
Overall, the manuscript by Cramm et al., provided a very interesting insight into the temporal patterns of microbial growth following thawing of Arctic permafrost through the use of DNA SIP. The manuscript provided good evidence to highlight taxon-specific growth dynamics of thawed soil microcosms over a prolonged time period, with clear evidence indicating that microbial communities shift over time, and that predatory bacterial taxons appear to benefit from this seasonal thawing process, which is likely to accelerate with a changing global climate. Overall, the manuscript was well-written and well-formatted, but there are a couple of methodological clarifications that should be addressed prior to the manuscript being accepted for publication.

We thank the reviewer for their positive, helpful and constructive comments and suggestions, all of which have been addressed below.

Major comments:
L129: What is the specific relevance of the 21 and 98 day timepoints? Do these timings correspond to specific atmospheric/climactic changes in the natural environment? As the soils were not used straight away after collection, it is unclear how the timepoints chosen align with the environmental temperature data shown in Figure 1D.

Soils were collected during winter, in a frozen state, and remained frozen after collection (including during storage and transport) until thawing to start the incubation.

The length of time that these soils (i.e. this specific site and depth) typically thaw in situ during the summer period is ~100 days. Thus, our incubation of 98 days is of comparable length to the duration of seasonal thawing in situ. The shorter 21-day incubation does not correspond to a climate feature and was included to identify earlier growing organisms from later growing organisms. Figure 1D provides site context during 2021, the year the soil samples were taken. We have added a sentence (lines 190-192) to clarify the how the length of our incubations relate to site characteristics.

L152: What was the rationale behind only performing the trace element analysis on soils collected on a different pit at a completely different time (March 2021 for microcosms, June 2022 for trace element analysis)? Whilst it may be possible that there are very limited changed in soil physicochemical properties over the course of 1.25 years, this cannot be definitively ruled out.

Thank you for this point. We agree. Unfortunately, samples for geochemical analyses were de-coupled from those for biological analyses as a result of logistical complexities of carrying out the fieldwork. We carried out the geochemical analyses to gain insight into the general geochemical context of the sample site. We have used this data solely for general reference to local soil chemistry (line 634) and made sure that our results and discussion do not rely on the chemical properties of the June 2022 soil pit. We also hope that including the chemical characterisation of this soil pit may serve as a helpful reference for soil characteristics at the Bayelva site on Svalbard.

L174-176: How many times over the incubation period was the headspace in the microcosms replaced? If the headspace wasn't replaced, this would likely lead to the vials becoming anoxic, particularly by the 98 day timepoint, which would likely culminate in shifts in microbial community composition. As the thawing soils would likely be exposed to a 'normal' atmosphere with regular gas flux, only adding in oxygen at the start of the incubation period, and using air without CH_4 or CO_2, may not accurately reflect the natural environment of the soils, and thus would alter the microbial community composition over the incubation period.

When gas was withdrawn from the microcosms for compositional analyses, we did not replace the headspace. A sentence has been added (line 216-217) to make this clear. 2 mL of headspace was withdrawn for the 98-day incubations over the course of the incubation, and 1.25 mL of headspace was withdrawn for the 21-day incubations. Our tests showed that measurements of CO_2 and CH_4 concentrations were not affected by headspace removal up to 4 mL of removed headspace. Oxygen concentrations measured in the vials confirmed that the microcosm headspace remained oxic during the incubation (Supplementary Figure 3B, lines 369). We also highlight in lines 689-691 that the redox environments change in situ owing to changing hydrology of the soil but that our results mirror community changes observed in situ during the summer (lines 700-702).

L444-467: Did you take a look into lower taxonomic ranks, or just phylum level data? This would be particularly helpful for ascribing functions to the different microbial communities, as generalising bacterial functions on a phylum level is not particularly informative.

We discuss lower taxonomic levels to explore growth dynamics of predatory clades (lines 531-532) and methanotrophic clades (lines 580-581; 590-591).

L511-546: Given the absence of methane in the headspace of the microcosms, and the lack of methanogenic species identified within the microbial communities, it seems likely that there would only be a negligible increase in methanotroph populations, unless they were mixotrophic? As mentioned before, a complete lack of methane in the microcosm headspace would also not be particularly representative of the natural environment, where methane would be present in trace amounts (similar to that of H2 and CO, as mentioned in L568).

We discuss the possibility that facultative methanotrophs were growing in our microcosms on other C sources in lines 602-606. We agree with this comment, especially that future studies focused on the microbial methane dynamics in Svalbard active layer soils should include trace CH_4 in the headspace.

Supplementary Figure 2: There is a huge spike in CH4 concentration in one of the day 21 microcosm replicates. Is there any explanation for this? And if so, how would this affect the community composition of that replicate compared to the others in the set? Additionally, is there seems to be a clear decrease in CO2 concentration for 3 out of 5 replicates for the 98 day microcosm, suggesting that these vials are no longer actively growing.

Thank you for this comment. The spike in CH_4 was a single measurement from a single vial, and it is not consistent with the overall trend. Moreover, it did not co-occur with a drop in oxygen, nor detectable methanogens in 16S rRNA gene amplicon libraries. Thus, we have cautiously disregarded it in our discussion. We indicate this in the discussion in lines 575-579. We discuss the decrease or levelling of CO_2 concentrations in 3 of 5 microcosms in lines 362 and have added a statement in line 364 highlighting possible ceased or slowed growth in these microcosms.

Minor comments:
L123-124: Missing word "to" - ".... glacial forefield soils that were sensitive to successional stage and local vegetation."

Thank you. We have fixed this sentence.

L416-420: In these lines, three bacterial phyla are mentioned, but only two numbers are quoted, so it is unclear which phyla correspond to which number as there is one missing.

Thank you for highlighting this. The two relative abundances listed correspond not to the abundances of each of the three phyla but rather the total combined relative abundance for these three phyla at 21 and 98 days. We have edited this sentence to make this clearer (line 468).

L546: Missing reference to manuscript Figure where data has arisen from.

ASV-level taxonomies are not presented in the figures throughout this manuscript; this data is presented only in the text for selected clades. The corresponding data presented at the phylum-level is visualized in Figures 3 and 4.

Supplementary Table 1: The layout of this table is confusing, as it currently suggests that trace elements were only measured in the 20-24cm depth profile, as opposed to the other metrics which were only measured in the 18-27cm depth profile? Additionally, was only one sample used to calculate the cell count and total DNA concentration, as there are no SDs reported?

Thank you for this comment. All of our data was generated from the soil core site, except for the trace element analyses using soils from a nearby pit. This is explained in response to an earlier reviewer comment, and we have clarified this in the Methods section of our revised manuscript. We have modified Supplementary Table 1 to make this clear. DNA concentration and cell counts were carried out in triplicate. We have modified the reporting of this data in Supplementary Table 1 to include the standard deviation based on these 3 measurements.

References

Bird, J. T., Tague, E. D., Zinke, L., Schmidt, J. M., Steen, A. D., Reese, B., Marshall, I. P. G., Webster, G., Weightman, A., Castro, H. F., Campagna, S. R. and Lloyd, K. G., 2019. Uncultured Microbial Phyla Suggest Mechanisms for Multi-Thousand-Year Subsistence in Baltic Sea Sediments. *mBio*, 10 (2), 1–15.

Grünberg, I., Groenke, B., Westermann, S. and Boike, J., 2024. Permafrost and Active Layer Temperature and Freeze/Thaw Timing Reflect Climatic Trends at Bayelva, Svalbard. *Journal of Geophysical Research: Earth Surface*, 129 (7), e2024JF007648.

Gudelj, I., Weitz, J. S., Ferenci, T., Claire Horner-Devine, M., Marx, C. J., Meyer, J. R. and Forde, S. E., 2010. An integrative approach to understanding microbial diversity: from intracellular mechanisms to community structure. *Ecology Letters*, 13 (9), 1073–1084.

Jørgensen, B. B. and Marshall, I. P. G., 2016. Slow Microbial Life in the Seabed. *Annual Review of Marine Science*, 8 (Volume 8, 2016), 311–332.

Nguyen, J., Fernandez, V., Pontrelli, S., Sauer, U., Ackermann, M. and Stocker, R., 2021. A distinct growth physiology enhances bacterial growth under rapid nutrient fluctuations. *Nature Communications*, 12 (1), 3662.

Richards, O. C. and Boyer, P. D., 1966. 18O Labeling of deoxyribonucleic acid during synthesis and stability of the label during replication. *Journal of Molecular Biology*, 19 (1), 109–119.

Sieradzki, E. T., Koch, B. J., Greenlon, A., Sachdeva, R., Malmstrom, R. R., Mau, R. L., Blazewicz, S. J., Firestone, M. K., Hofmockel, K. S., Schwartz, E., Hungate, B. A. and Pett-Ridge, J., 2020. Measurement Error and Resolution in Quantitative Stable Isotope Probing: Implications for Experimental Design. *mSystems*, 5 (4).

Trejos-Espeleta, J. C., Marin-Jaramillo, J. P., Schmidt, S. K., Sommers, P., Bradley, J. A. and Orsi, W. D., 2024. Principal role of fungi in soil carbon stabilization during early pedogenesis in the high Arctic. *Proceedings of the National Academy of Sciences*, 121 (28), e2402689121.

Re: mSystems00738-25R1 (Seasonal thawing of high Arctic soils triggers selective microbial growth and predation)

Dear Dr. Margaret Anne Cramm:

Thank you for the privilege of reviewing your work. Below you will find my comments, instructions from the mSystems editorial office, and the reviewer comments.

Please return the manuscript within 60 days; if you cannot complete the modification within this time period, please contact me. If you do not wish to modify the manuscript and prefer to submit it to another journal, notify me immediately so that the manuscript may be formally withdrawn from consideration by mSystems.

Revision Guidelines
To submit your modified manuscript, log into the submission site at https://msystems.msubmit.net/cgi-bin/main.plex. Go to Author Tasks and click the appropriate manuscript title to begin. The information you entered when you first submitted the paper will be displayed; update this as necessary. Note the following requirements:

• Upload point-by-point responses to the issues raised by the reviewers in a file named "Response to Reviewers," NOT in your cover letter.
• Upload a compare copy of the manuscript (without figures) as a "Marked-Up Manuscript" file.
• Upload a clean .DOC/.DOCX version of the revised manuscript and remove the previous version.
• Each figure must be uploaded as a separate, editable, high-resolution file (TIFF or EPS preferred), and any multipanel figures must be assembled into one file.
• Any supplemental material intended for posting by ASM should be uploaded with their legends separate from the main manuscript. You can combine all supplemental material into one file (preferred) or split it into a maximum of 10 files with all associated legends included.

For complete guidelines on revision requirements, see our Submission and Review Process webpage. Submission of a paper that does not conform to guidelines may delay acceptance of your manuscript.

Data availability: ASM policy requires that data be available to the public upon online posting of the article, so please verify all links to sequence records, if present, and make sure that each number retrieves the full record of the data. If a new accession number is not linked or a link is broken, provide mSystems production staff with the correct URL for the record. If the accession numbers for new data are not publicly accessible before the expected online posting of the article, publication may be delayed; please contact production staff (mSystems@asmusa.org) immediately with the expected release date.

Publication Fees: For information on publication fees and which article types are subject to charges, visit our website. If your manuscript is accepted for publication and any fees apply, you will be contacted separately about payment during the production process; please follow the instructions in that e-mail. Arrangements for payment must be made before your article is published.

ASM Membership: Corresponding authors may join or renew ASM membership to obtain discounts on publication fees. Need to upgrade your membership level? Please contact Customer Service at Service@asmusa.org.

The ASM Journals program strives for constant improvement in our submission and publication process. Please tell us how we can improve your experience by taking this quick Author Survey.

Thank you for submitting your paper to mSystems.

Sincerely,
Emily Graham
Editor
mSystems

Reviewer #1 (Comments for the Author):

Overall comments
The authors have worked to address the large bulk of my concerns. There are only three issues that must be corrected. These issues concern either the accuracy of results reporting or interpretation and cannot be ignored.

Specific comments

L380: This statement on growing community composition remains incorrect given the data shown, and the responses to reviewers from the authors. My understanding is that growing communities are compositionally distinct while total communities are similar. Is this statement meant to correspond to total community composition?

L548-552: This sentence has not been amended to sufficiently clarify the authors meaning from the previously. For methane oxidation, the statement that "up to half of the organisms" grew is ambiguous. The issue was not on saying "roughly half the organisms" or "up to half the organisms" but on the use of the word "organisms". To me, "organism" functions similarly to "taxon" here -- it implies that the cells of one taxon grew while the cells of another taxon did not. From their response, the authors correctly communicate to reviewers that this conclusion pertains to aggregate DNA labelling; this needs to be in the main text as well. Figure 5 directly indicates that roughly half of the DNA material containing pmoA genes has incorporated 18O which suggests that a corresponding half of the cells sampled underwent replication during the incubation period.

L491-493: I still think this statement is confusing and misleading. The previous sentence show that rapid response traits should be linked to rapid growth at the earlier timepoint -- I agree. By contrast, once the initial food sources are depleted, we should see slow-growing organisms -- I agree. But the authors include "changing environments" with this statement. Why? Changing environments corresponds to the fast responders per their previous sentence. Also the, Nguyen paper discusses population growth response to fluctuating conditions -- it does not identify trait trade-offs or fitness response to a fluctuating environment. I recommend removing the "changing environments" and Nguyen paper. This change would clarify, as I understand it, the authors' point.

Reviewer #2 (Comments for the Author):

Overall:
Overall, the manuscript by Cramm et al., provided a very interesting insight into the temporal patterns of microbial growth and community structuring following thawing of Arctic permafrost through the use of permafrost microcosms and DNA SIP. The revised manuscript provided good clarifications to the original text to support the notion of taxon-specific growth dynamics of thawed soil microcosms over a prolonged time period, with clear evidence indicating that microbial communities shift over time, and that predatory bacterial taxons appear to benefit from this seasonal thawing process, which is likely to accelerate with a changing global climate. The revised manuscript has benefited distinctly from the specific clarification of various methodological subsections which have clarified a number of key discrepancies in the previous manuscript, as well as the toning down of language around the findings due to experimental limitations surrounding replication, hydrology and lack of trace gases within the microcosm headspace. Whilst the low number of replicates slightly dampens the impact of the findings, the difficulties of sampling in these remote Arctic regions should be taken into consideration. The overall importance and scope of manuscript's findings have become much clearer now from the inclusion of previous reviewer suggestions.

Minor Comments:
Figure 3: What is the medium blue bar in the first replicate microcosm in Figure 3B (about halfway down, under the Chloroflexi)? It doesn't appear to have a corresponding coloured dot in the key present. It also seems to only be in that replicate and in the pre-thaw sample.

L560-563: The pmoA primers that you used were highly specific, and unable to amplify divergent pmoA-like genes such as from Verrucomicrobiota, however, as there was an increase in this phylum in the 98-day SIP microbial communities, it seems remiss to not have included an additional qPCR to see if the pmoA-like genes associated with Verrucomicrobiota/Methylomicrobilota were present in the samples or not.

L593-L595: While I agree that there are likely low concentrations of some of the trace metals, the amounts of most of them listed in Table S1 seem to be within the normal range for soil systems. What was the pH of these soils? As this could affect the bioavailability of the trace elements more so than just concentration.

<u>Reviewer Comments:</u>

Manuscript number: mSystems00738-25R1
Article title: Seasonal thawing of high Arctic soils triggers selective microbial growth and predation
Article type: Original research

Overall:

Overall, the manuscript by Cramm et al., provided a very interesting insight into the temporal patterns of microbial growth and community structuring following thawing of Arctic permafrost through the use of permafrost microcosms and DNA SIP. The revised manuscript provided good clarifications to the original text to support the notion of taxon-specific growth dynamics of thawed soil microcosms over a prolonged time period, with clear evidence indicating that microbial communities shift over time, and that predatory bacterial taxons appear to benefit from this seasonal thawing process, which is likely to accelerate with a changing global climate. The revised manuscript has benefited distinctly from the specific clarification of various methodological subsections which have clarified a number of key discrepancies in the previous manuscript, as well as the toning down of language around the findings due to experimental limitations surrounding replication, hydrology and lack of trace gases within the microcosm headspace. Whilst the low number of replicates slightly dampens the impact of the findings, the difficulties of sampling in these remote Arctic regions should be taken into consideration. The overall importance and scope of manuscript's findings have become much clearer now from the inclusion of previous reviewer suggestions.

Minor Comments:

Figure 3: What is the medium blue bar in the first replicate microcosm in Figure 3B (about halfway down, under the Chloroflexi)? It doesn't appear to have a corresponding coloured dot in the key present. It also seems to only be in that replicate and in the pre-thaw sample.

L560-563: The pmoA primers that you used were highly specific, and unable to amplify divergent pmoA-like genes such as from Verrucomicrobiota, however, as there was an increase in this phylum in the 98-day SIP microbial communities, it seems remiss to not have included an additional qPCR to see if the pmoA-like genes associated with Verrucomicrobiota/Methylomicrobilota were present in the samples or not.

L593-L595: While I agree that there are likely low concentrations of some of the trace metals, the amounts of most of them listed in Table S1 seem to be within the normal range for soil systems. What was the pH of these soils? As this could affect the bioavailability of the trace elements more so than just concentration.

Response to editor and reviewers' comments on 'Seasonal thawing of high Arctic soils triggers selective microbial growth and predation (mSystems00738-25)

EDITOR

Thank you for the privilege of reviewing your work. Below you will find my comments, instructions from the mSystems editorial office, and the reviewer comments.

We would like to thank the editor and reviewers for their constructive comments and suggestions on our revised submission, which have helped further improve the manuscript. We have addressed all the suggestions raised below.

Please note that the line numbers we quote in our response are correct according to the "marked-up" version of the document when track changes are visible to the reader (i.e. "All Markup" is selected in the Review pane). The reviewer's comments are listed in bold below.

Reviewer #1 (Comments for the Author):

Overall comments
The authors have worked to address the large bulk of my concerns. There are only three issues that must be corrected. These issues concern either the accuracy of results reporting or interpretation and cannot be ignored.

Thank you for your comments which have greatly improved the clarity of this paper. We thank you for participating in this second round of reviews. We have addressed your specific comments below.

Specific comments

L380: This statement on growing community composition remains incorrect given the data shown, and the responses to reviewers from the authors. My understanding is that growing communities are compositionally distinct while total communities are similar. Is this statement meant to correspond to total community composition?

Thank you for highlighting this. We agree with your observations and have corrected the manuscript accordingly (line 393-394). The sentence now reads: The overall composition of the total microbial communities present at the 'early' and 'late' incubation timepoints were similar.

L548-552: This sentence has not been amended to sufficiently clarify the authors meaning from the previously. For methane oxidation, the statement that "up to half of the organisms" grew is ambiguous. The issue was not on saying "roughly half the organisms" or "up to half the organisms" but on the use of the word "organisms". To me, "organism" functions similarly to "taxon" here -- it implies that the cells of one taxon grew while the cells of another taxon did not. From their response, the authors correctly communicate to reviewers that this conclusion pertains to aggregate DNA labelling; this needs to be in the main text as well. Figure 5 directly indicates that roughly half of the DNA material containing pmoA genes has incorporated 18O which

suggests that a corresponding half of the cells sampled underwent replication during the incubation period.

Thank you for clarifying this comment further. We agree and have edited the text to read as follows:

The aggregate ^{18}O-labelled *pmoA* gene DNA was of similar abundance to the aggregate non-labelled *pmoA* gene DNA, indicating that up to half of the *pmoA* containing cells grew during the 98 days following thaw. (lines 567-572)

L491-493: I still think this statement is confusing and misleading. The previous sentence show that rapid response traits should be linked to rapid growth at the earlier timepoint -- I agree. By contrast, once the initial food sources are depleted, we should see slow-growing organisms -- I agree. But the authors include "changing environments" with this statement. Why? Changing environments corresponds to the fast responders per their previous sentence. Also the, Nguyen paper discusses population growth response to fluctuating conditions -- it does not identify trait trade-offs or fitness response to a fluctuating environment. I recommend removing the "changing environments" and Nguyen paper. This change would clarify, as I understand it, the authors' point.

Thank you. These are great points. We have removed the “changing environments” and the Nguyen paper from this sentence. The sentence now reads: “Slow growth is also favoured by organisms residing in harsh environments.” (line 480).

<u>Reviewer #2 (Comments for the Author):</u>

Overall:
Overall, the manuscript by Cramm et al., provided a very interesting insight into the temporal patterns of microbial growth and community structuring following thawing of Arctic permafrost through the use of permafrost microcosms and DNA SIP. The revised manuscript provided good clarifications to the original text to support the notion of taxon-specific growth dynamics of thawed soil microcosms over a prolonged time period, with clear evidence indicating that microbial communities shift over time, and that predatory bacterial taxons appear to benefit from this seasonal thawing process, which is likely to accelerate with a changing global climate. The revised manuscript has benefited distinctly from the specific clarification of various methodological subsections which have clarified a number of key discrepancies in the previous manuscript, as well as the toning down of language around the findings due to experimental limitations surrounding replication, hydrology and lack of trace gases within the microcosm headspace. Whilst the low number of replicates slightly dampens the impact of the findings, the difficulties of sampling in these remote Arctic regions should be taken into consideration. The overall importance and scope of manuscript's findings have become much clearer now from the inclusion of previous reviewer suggestions.

Minor Comments:
Figure 3: What is the medium blue bar in the first replicate microcosm in Figure 3B (about halfway down, under the Chloroflexi)? It doesn't appear to have a corresponding coloured dot in the key present. It also seems to only be in that replicate and in the pre-thaw sample.

The light blue bar in the first replicate microcosm in Figure 3B corresponds to the relative abundance of a single ASV of the phylum Dependentiae, of order Candidatus Babeliales, an intracellular protist parasite. This phylum was not identified in the qSIP analyses (Figure 3C), likely because its presence was not reproducible across the replicate microcosms – this ASV would need to be present in both the 18O-amended microcosm and the control microcosm to be identified by qSIP.

We have added this Phylum to the key of Figure 3C.

L560-563: The pmoA primers that you used were highly specific, and unable to amplify divergent pmoA-like genes such as from Verrucomicrobiota, however, as there was an increase in this phylum in the 98-day SIP microbial communities, it seems remiss to not have included an additional qPCR to see if the pmoA-like genes associated with Verrucomicrobiota/Methylomicrobilota were present in the samples or not.

Our 16S rRNA gene amplicon data suggests that no known methanotrophic members of the Verrucomicrobiota or Methylomirabilota grew in our microcosms. None of the Verrucomicrobiota ASVs that were 18O-labelled were affiliated to the methanotrophic genera *Methylacidiphilum* or *Methylacidimicrobium* (identified by Schmitz et al.(2021)). Also, the Methylomirabilota ASVs that were 18O-labelled were all affiliated to the non-methanotrophic Order Rokubacteriales.

We have included a summary of the above information in lines 577-579 to clarify whether the Verrucomicrobiota or Methylomirabilota ASVs growing in our microcosms may be methanotrophic.

L593-L595: While I agree that there are likely low concentrations of some of the trace metals, the amounts of most of them listed in Table S1 seem to be within the normal range for soil systems. What was the pH of these soils? As this could affect the bioavailability of the trace elements more so than just concentration.

Thanks for this insight. We have added the soil pH (6.05) to Table S1. We have also removed our suggestion of the low trace metals restraining microbial growth and instead suggested physico-chemical factors including pH may affect trace metal bioavailability in lines 622-625.

<u>References</u>

Schmitz, R. A., Peeters, S. H., Versantvoort, W., Picone, N., Pol, A., Jetten, M. S. M. and Op den Camp, H. J. M., 2021. Verrucomicrobial methanotrophs: ecophysiology of metabolically versatile acidophiles. *FEMS Microbiology Reviews*, 45 (5), fuab007.

Re: mSystems00738-25R2 (Seasonal thawing of high Arctic soils triggers selective microbial growth and predation)

Dear Dr. Margaret Anne Cramm:

Thank you for the privilege of reviewing your work. Below you will find my comments, instructions from the mSystems editorial office, and the reviewer comments.

Please add the Data Availability section to the manuscript.
https://journals.asm.org/journal/msystems/article-types

Please return the manuscript within 60 days; if you cannot complete the modification within this time period, please contact me. If you do not wish to modify the manuscript and prefer to submit it to another journal, notify me immediately so that the manuscript may be formally withdrawn from consideration by mSystems.

Revision Guidelines
To submit your modified manuscript, log into the submission site at https://msystems.msubmit.net/cgi-bin/main.plex. Go to Author Tasks and click the appropriate manuscript title to begin. The information you entered when you first submitted the paper will be displayed; update this as necessary. Note the following requirements:

• Upload point-by-point responses to the issues raised by the reviewers in a file named "Response to Reviewers," NOT in your cover letter.
• Upload a compare copy of the manuscript (without figures) as a "Marked-Up Manuscript" file.
• Upload a clean .DOC/.DOCX version of the revised manuscript and remove the previous version.
• Each figure must be uploaded as a separate, editable, high-resolution file (TIFF or EPS preferred), and any multipanel figures must be assembled into one file.
• Any supplemental material intended for posting by ASM should be uploaded with their legends separate from the main manuscript. You can combine all supplemental material into one file (preferred) or split it into a maximum of 10 files with all associated legends included.

For complete guidelines on revision requirements, see our Submission and Review Process webpage. Submission of a paper that does not conform to guidelines may delay acceptance of your manuscript.

Data availability: ASM policy requires that data be available to the public upon online posting of the article, so please verify all links to sequence records, if present, and make sure that each number retrieves the full record of the data. If a new accession number is not linked or a link is broken, provide mSystems production staff with the correct URL for the record. If the accession numbers for new data are not publicly accessible before the expected online posting of the article, publication may be delayed; please contact production staff (mSystems@asmusa.org) immediately with the expected release date.

Publication Fees: For information on publication fees and which article types are subject to charges, visit our website. If your manuscript is accepted for publication and any fees apply, you will be contacted separately about payment during the production process; please follow the instructions in that e-mail. Arrangements for payment must be made before your article is published.

ASM Membership: Corresponding authors may join or renew ASM membership to obtain discounts on publication fees. Need to upgrade your membership level? Please contact Customer Service at Service@asmusa.org.

The ASM Journals program strives for constant improvement in our submission and publication process. Please tell us how we can improve your experience by taking this quick Author Survey.

Thank you for submitting your paper to mSystems.

Sincerely,
Emily Graham
Editor
mSystems

Response to editor and reviewers' comments on 'Seasonal thawing of high Arctic soils triggers selective microbial growth and predation (mSystems00738-25)

<u>EDITOR</u>

Please add the Data Availability section to the manuscript.

We would like to thank the editor for helping to further improve this manuscript. We have added a Data Availability section to the manuscript under the section title "Data Availability Statement".

Re: mSystems00738-25R3 (Seasonal thawing of high Arctic soils triggers selective microbial growth and predation)

Dear Dr. Margaret Anne Cramm:

Your manuscript has been accepted, and I am forwarding it to the ASM production staff for publication. Your paper will first be checked to make sure all elements meet the technical requirements. ASM staff will contact you if anything needs to be revised before copyediting and production can begin. Otherwise, you will be notified when your proofs are ready to be viewed.

Data Availability: ASM policy requires that data be available to the public upon online posting of the article, so please verify all links to sequence records, if present, and make sure that each number retrieves the full record of the data. If a new accession number is not linked or a link is broken, provide production staff with the correct URL for the record. If the accession numbers for new data are not publicly accessible before the expected online posting of the article, publication may be delayed; please contact ASM production staff immediately with the expected release date.

Publication Fees: For information on publication fees and which article types have charges, please visit our website. We have partnered with Copyright Clearance Center (CCC) to collect author charges. If fees apply to your paper, you will receive a message from no-reply@copyright.com with further instructions. For questions related to paying charges through RightsLink, please contact CCC at ASM_Support@copyright.com or toll free at +1-877-622-5543. CCC makes every attempt to respond to all emails within 24 hours.

ASM Membership: Corresponding authors may join or renew ASM membership to obtain discounts on publication fees. Need to upgrade your membership level? Please contact Customer Service at Service@asmusa.org.

Embargo Policy: A press release may be issued as soon as the manuscript is posted on the mSystems Latest Articles webpage. The corresponding author will receive an email with the subject line "ASM Journals Author Services Notification" when the article is available online.

Cover Image Submissions: If you would like to submit a potential Cover Image, please email a file and a short legend to msystems@asmusa.org. Please note that we can only consider images that (i) the authors created or own and (ii) have not been previously published. By submitting, you agree that the image can be used under the same terms as the published article. Image File requirements: TIF/EPS, 7.5 inches wide by 8.25 inches tall (at least 2,250 pixels wide by 2,475 pixels tall), minimum 300 dpi resolution (600 dpi preferred), RGB, and no figure elements, e.g., arrows or panel labels. The legend should be a short description of the image, 1-2 sentences recommended. Please download and use this interactive template in Adobe to ensure that your proposed cover image meets our size requirements (https://journals.asm.org/pb-assets/pdf-text-excel-files/ASM-Interactive-Sizing-Cover-Template-1715689791.pdf).

Thank you for submitting your paper to mSystems.

Sincerely,
Emily Graham
Editor
mSystems
